# Connectivity-Driven Electronic Structure and Charge Separation in Morpholinium-Based Bi^3+^/Sb^3+^ Halides

**DOI:** 10.1021/acs.inorgchem.6c02197

**Published:** 2026-07-02

**Authors:** Tamara J. Bednarchuk, Magdalena N. Rowińska, Oleksandr Korolevych, Dagmara Stefańska, Anna Gagor

**Affiliations:** Institute of Low Temperature and Structure Research, 215275 Polish Academy of Sciences, Okólna 2, 50-422 Wrocław, Poland

## Abstract

Compositional mixing in organic–inorganic metal-halide double perovskite related materials leads to diverse structural motifs with distinct polyhedral connectivity. In A_2_MM′X_6_ compounds, the organic A-site cation and alkali-metal M ion play a key role in determining the connectivity of M′X_6_ (M′ = Bi^3+^, Sb^3+^) octahedra and electronic properties. Here, we report four morpholinium (MOR)-based halides: (MOR)_2_CsBiCl_6_ (**1**), (MOR)_2_CsSbCl_6_ (**2**), (MOR)_2_KBiCl_6_ (**3**), and (MOR)_2_RbSbI_6_ (**4**), characterized by single-crystal X-ray diffraction and DFT calculations. Despite identical composition, the compounds adopt distinct architectures: **1** is centrosymmetric with nearly regular octahedra, **2** crystallizes in a polar space group with distorted SbCl_6_ units, **3** forms a 1D edge-sharing framework, whereas **4** adopts a 3D framework with different connectivity. Effective-mass calculations reveal anisotropic charge transport in **1** and **2**, with higher electron than hole mobility and spatial separation of band-edge charge densities in **1**, consistent with a direct Z-scheme heterojunction. Photoluminescence at 80 K reveals two emission bands (415/440 nm) with distinct recombination mechanisms arising from the band gap offset between inequivalent BiCl_6_ units. Large Cl···Cl separations in **3** suppress orbital overlap and limit charge mobility. These results highlight the roles of cation size, lone-pair activity, and halide polarizability in directing structure and electronic behavior.

## Graphical abstract

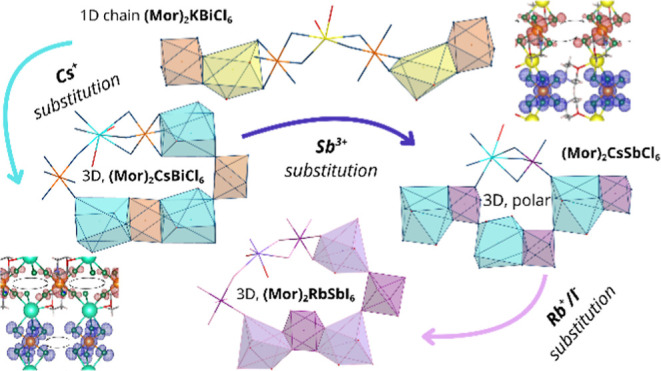

## 1 Introduction

Over the past decades, hybrid perovskite materials with the general formula ABX_3_ (where A is an organic ammonium cation, B is a divalent metal such as Pb^2+^, Sn^2+^, Ge^2+^, and X is a halide anion: Cl^–^, Br^–^, or I^–^) have attracted considerable attention owing to their exceptional structural, electronic, and optical versatility. Their outstanding charge-transport properties, tunable band gaps, and facile solution-based synthesis make them highly promising for a wide range of optoelectronic and photovoltaic applications.^1–7^ To address the environmental concerns associated with Pb^2+^-containing perovskites, research efforts have increasingly turned among others toward bismuth­(III)- and antimony­(III)-based organic–inorganic hybrids, which preserve many of the favorable optoelectronic features of lead analogs while offering greater chemical stability and reduced toxicity.^8–11^ Compositional tuning within this materials family has led to the development of halide double perovskite related compounds with the general formula A_2_MM′X_6_. In these structures, the A-site is typically occupied by a monovalent cation, analogous to conventional ABX_3_ perovskites, while the B-site alternates between monovalent (M = Na^+^, K^+^, Rb^+^, Ag^+^) and trivalent (M′ = Bi^3+^, Sb^3+^, In^3+^) metal centers, and the X-site corresponds to a halide anion. Depending on the specific combination of metal cations and halides, these materials exhibit an exceptional diversity of structural dimensionalities, connectivity types, and electronic behaviors— from wide-gap insulators to semiconductors with band edges well-suited for optoelectronic applications.^12–16^

Our previous study focused on the organic–inorganic hybrid (Pip)_2_[KBiBr_6_] (Pip = piperidinium),^17^ which features alternating chains of face-sharing BiBr_6_ octahedra and KBr_6_ polyhedra separated by Pip^+^ counterions. This compound represents a rare example of a molecular hybrid halide that undergoes a ferroelastic phase transition at near-room temperatures. The structural transformation is driven by the partial ordering of Pip^+^ cations, the reorganization of bifurcated N–H···Br hydrogen bonds, and the accompanying rearrangement of the inorganic framework. Together, these processes highlight the crucial role of hydrogen bonding and weak van der Waals halogen···halogen interactions between bismuth halide octahedra in imparting structural flexibility and stabilizing hybrid halide architectures.

The present study explores new members of morpholinium alkali bismuth and antimony halides, integrating synthesis, structural characterization, and DFT analysis to elucidate how cation coordination and polyhedral connectivity influence the resulting architectures and electronic structure. Morpholinium (MOR), possessing both NH and C=O donor sites, offers a unique opportunity to form simultaneous hydrogen-bonding and coordination interactions with halide frameworks. Coordination of the C=O group to the monovalent metal center transforms the conventional [MX_6_] octahedra into [MX_*n*_O_2_] polyhedra, thereby redefining the connectivity between [MX_*n*_O_2_] and [M′X_6_] units and giving rise to frameworks of varying dimensionality (Scheme 1).

**Scheme 1:**
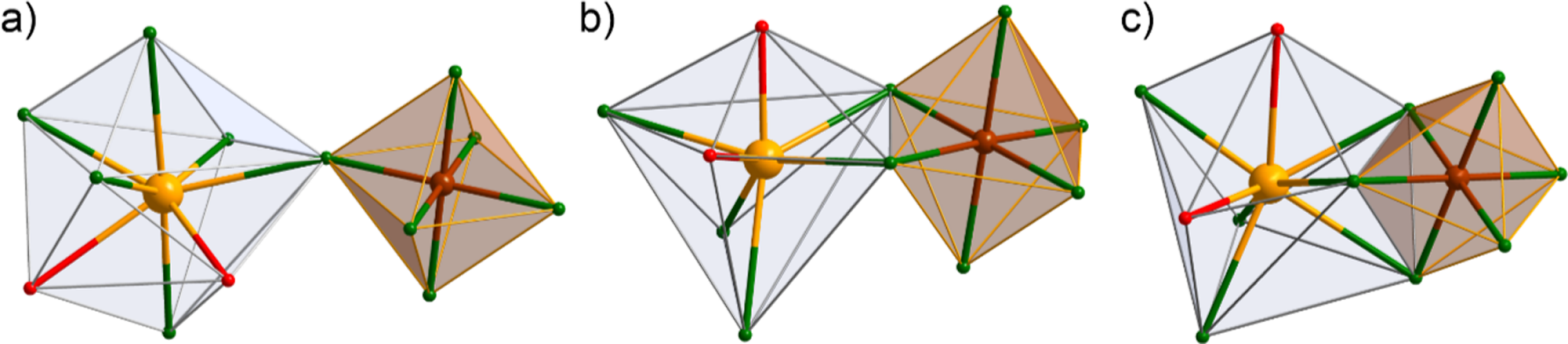
Schematic Illustration of the Three Connectivity Modes Observed within the Series: (a) Corner-Sharing, (b) Edge-Sharing, and (c) Face-Sharing between [MX_*n*_O_2_] Polyhedra and [M′X_6_] Octahedra

Previous studies on morpholinium-based hybrid halides have primarily focused on bromide and iodide systems, which served as structural and synthetic reference points for the present work.^15^ In that study, 15 new Bi-based double perovskite related halides with the general formula of A_2_BBiX_6_ (A = 1-methylpyrrolidin-1-ium-3-ol, morpholinium, 4-methylmorpholinium, ethanolammonium, methyl-2-hydroxyethylammonium cations; B = K, Rb; X = Br, I) were synthesized. Among these, four morpholinium-based compounds were reported, displaying distinct dimensionalities depending on the halide and alkali metal combination. The K-based analogs, (MOR)_2_KBiBr_6_ and (MOR)_2_KBiI_6_, adopt one-dimensional (1D) chain structures crystallizing in the monoclinic space groups *C*2/*c* (centrosymmetric) and *C*2 (noncentrosymmetric), respectively. Substitution of K^+^ by the larger Rb^+^ cation leads to increased connectivity: (MOR)_2_RbBiBr_6_·H_2_O forms two-dimensional (2D) layers in *Pmmn*, while (MOR)_2_RbBiI_6_ develops a three-dimensional (3D) framework of tetrameric units in *C*2/*c* space group.

Motivated by these findings, we extended this chemistry to the corresponding chloride analogues and antimony-substituted compounds to systematically evaluate how variations in halide size, polarizability, and *B*-site cation character (Bi^3+^ vs Sb^3+^) influence framework dimensionality, octahedral distortion, and overall structural stability.

Furthermore, two of the morpholinium-based halides described in this study - those containing K^+^ (**3**) and Rb^+^ (**4**) cations - were independently reported during the course of our work.^18,19^ For completeness and consistency, these compounds are also discussed here using our own diffraction data and/or DFT calculations, allowing a unified structural and electronic comparison across the entire series.

## 2 Experimental Section

### 2.1 Synthesis

The single crystals were obtained by mixing stoichiometric amounts of morpholine (≥99%), KCl/CsCl/RbI (pure WARCHEM), and Bi_2_O_3_/Sb_2_O_3_ (≥99.90%, Acros Organics) (4:2:1) in 5 mL of concentrated hydrochloric acid or hydroiodic acid (57%, Acros Organics). After slow evaporation of the solution, clear crystals appeared.

### 2.2 Optical Measurements

The RT diffuse reflectance spectra of powdered samples were obtained using the Agilent Cary 5000 spectrophotometer in the wavelength range of 240 to 800 nm, with a step size of 0.25 nm. Based on the reflectance spectrum, the energy band gap (*E*_g_) was estimated using the Kubelka–Munk relation^20^ and Tauc modification^21^ using *n* = 1/2 for direct (compound **3**) and *n* = 2 for indirect (compounds **1**, **2**) transition bandgaps, respectively. Time-resolved PL spectra and decay curves were measured using a femtosecond laser (Coherent Model Libra) coupled with a streak camera. The temperature of the sample was controlled using Linkam THMS 600 heating/freezing stage. Photoluminescence excitation spectra (PLE) and Photoluminescence (PL) were registered using a FLS1000 spectrofluorometer (Edinburgh Instruments, Livingston, UK) with 450 W xenon lamp.

### 2.3 X-ray Analysis

The single-crystal X-ray diffraction was collected on an Oxford Diffraction Xcalibur four-circle diffractometer with a graphite-monochromated Mo *K*α (λ = 0.71073 Å) radiation and an Atlas CCD detector. Data collection and reduction were performed using the CrysAlisPro programs.^22^ The latter software was also used to define the crystal shape and perform the absorption correction. The structures were solved by SHELXT methods^23^ with the Olex^2^ graphical user interface,^24^ and all non-hydrogen atoms were refined anisotropically by the least-squares technique on weighted F^2^ using SHELXL crystallographic software package.^25^ The hydrogen atoms attached to the C and N atoms were placed in idealized positions. The crystal structure of compound **2** was refined as an inversion twin. The refined Flack parameter of 0.518(15) indicates that the macroscopic crystal is twinned by inversion in an approximately 1:1 ratio of enantiomorphic domains. Attempts to refine the model in any centrosymmetric space group did not yield a satisfactory structural description, and PLATON symmetry analysis revealed no missed or higher symmetry, confirming *Pca*2_1_ as the correct noncentrosymmetric space group. The unit cell of compound **3** (monoclinic space group *I*2/*a*) was chosen with the β angle close to 90°. The software DIAMOND 3.2 ^26^ was used to create graphic representations of the crystal structure.

Powder XRD patterns were measured in the reflection mode on an X’Pert PRO X-ray diffraction system equipped with a PIXcel ultrafast line detector and Soller slits for Cu Kα radiation (λ = 1.5418 Å).

To investigate the contributions of intermolecular interactions, present in the structures of the title compounds, Hirshfeld surfaces and 2D fingerprint plots for the investigated crystals were calculated and analyzed using Crystal Explorer Ver. 17.5 software package.^27^ Calculations of polyhedral deformation indices were performed by Vesta.^28^

### 2.4 Computational (DFT) Studies

First-principles calculations based on density functional theory (DFT) were carried out using the QUANTUM ESPRESSO package (version 6.8).^29,30^ The exchange–correlation energy was treated within the generalized gradient approximation (GGA) using the Perdew–Burke–Ernzerhof (PBE) functional.^31^ Scalar-relativistic and fully relativistic pseudopotentials were employed within the projector augmented-wave (PAW) method, as generated by A. Dal Corso.^32^ Plane-wave kinetic energy cutoffs were set to 40 Ry for the wave functions and 400 Ry for the charge density. The Brillouin zone was sampled using an 8 × 8 × 8 Γ-centered Monkhorst–Pack grid.^33^ Self-consistent field (SCF) calculations were converged to an energy threshold of 10^–8^ Ry. Nonself-consistent (NSCF) calculations were performed using the tetrahedron method for accurate electronic structure evaluation.^34^ High-symmetry *k*-point paths used for the band structure calculations were generated using the SeeK-path tool.^35^ The Brillouin zone paths for all compounds are listed in Supporting Information. A comprehensive overview of all DFT-related methodological details, together with the complete set of band-structure, PDOS, effective-mass and charge-density analyses, is provided in the Supporting Information (Table S1; Figures S1–S5).

The effective masses of charge carriers were obtained using the parabolic approximation of the band dispersion, determined from the second derivative of the energy *E*(*k*) with respect to the crystal momentum *k*, according to $\frac{1}{m^{*}}=\frac{1}{\hbar^{2}}\frac{d^{2}\left(E\left(k\right)\right)}{dk^{2}}$ .

DFT calculations were performed for all four compounds based on experimental crystal structures. To validate the use of experimental crystal structures as the basis for DFT calculations across all four compounds, a test geometry optimization was carried out for (MOR)_2_CsBiCl_6_, see details in Supporting Information, DFT calculations section. A comparison of the scalar relativistic band structures obtained with and without structural relaxation (Figure S2) revealed that the resulting band gaps differed only negligibly between the two approaches. For the first time the calculations were performed for two newly synthesized morpholinium-based Bi^3+^/Sb^3+^ chlorides, (MOR)_2_CsBiCl_6_ (**1**) and (MOR)_2_CsSbCl_6_ (**2**), together with the recently reported analogs (MOR)_2_KBiCl_6_ (**3**)^18^ and (MOR)_2_RbSbI_6_ (**4**).^19^ To the best of our knowledge, no first-principles study has been reported for **3**. As far as compound **4** is concerned, the DFT calculations have been already provided using similar methodology.^19^ For the sake of completeness, we report our results for **4** in Supporting Information (Figure S5) in the DFT calculations section.

For bismuth halides, functionals such as GGA/PBE (e.g., for Cs_3_Bi_2_I_9_ and MA_3_Bi_2_I_9_)^36^ or GGA/PBE + SOC (e.g., for Cs_2_NaBiI_6_)^37^ have been shown in prior studies to provide a reasonable and reliable description of the electronic structure. At the same time, the behavior of bismuth halides differs significantly from that of their lead-based analogs. For lead halides, it is well established that GGA/PBE underestimates band gaps. Hybrid functionals such as HSE06,^38^ typically combined with SOC due to the strong influence of Pb 6p orbitals,^39^ are more commonly employed. The direct transfer of approach methodology between those systems is not straightforward. The electronic structure of bismuth halides involves additional complexities, and in such cases the inclusion of a fraction of nonlocal Hartree–Fock exchange may lead to an overestimation of certain properties, potentially resulting in larger deviations rather than improved accuracy. Trial calculation and previous report clearly indicated this behavior. For instance, in the case of Cs_2_NaBiI_6_,^37^ the experimentally determined band gap is approximately 1.66 eV,^40^ whereas GGA/PBE predicts a value of 2.23 eV. Inclusion of spin–orbit coupling within GGA/PBE yields a band gap of 1.68 eV, in excellent agreement with experiment, while HSE06 + SOC substantially overestimates the gap at 2.43 eV.^37^ These results are consistent with our previous reports on related bismuth-based hybrids, such as (piperidinium)_2_KBiCl_6_ and (piperidinium)_2_KBiBr_6_,^17^ where GGA/PBE + SOC leads to improvement of the calculated band gaps to the experimental value. A similar trend has also been observed for (MEPRD)_2_RbBiBr_6_ and (MEPRD)_2_KBiBr_6_ (MEPRD = 1-methylpyrrolidin-1-ium-3-ol).^16^ Generally, the correct choice of a DFT method should be based on the latest developments in ab initio approaches, as demonstrated for Ag-based halide double perovskites,^41^ and should include trial simulations rather than relying on a straightforward application of a single selected computational methodology.

Here, we present a connectivity-resolved DFT analysis that rationalizes the experimental absorption edge and places it within the broader family of morpholinium-based Bi^3+^/Sb^3+^ halides.

## 3 Results and Discussion

### 3.1 Crystal and Molecular Structures of **1** and **2**

Single-crystal X-ray diffraction (SC-XRD) analyses confirmed that compounds **1** and **2** adopt the general formula (C_4_H_10_NO)_2_MM′X_6_, but crystallize in distinct structural types: monoclinic *P*2_1_/*c* (**1**) and orthorhombic *Pca*2_1_ (**2**). For a comprehensive comparison with the literature-reported analogs (compounds **3** and **4**), structural models based on our own diffraction data were employed to ensure full consistency across the data set. In the case of compound **3**, refinement was performed in the nonstandard space group *I*2/*a*, where the β angle is close to 90° (92.78(3)° in our model), in contrast to the standard setting *C*2/*c* with β ≈ 112° used by Li et al.^18^ For compound **4**, the structure was determined at room temperature to enable direct comparison with compounds **1–3**, since the previously published model had been refined at 120 K.

Crystal data, data collection, and refinement parameters are summarized in Table 1, and hydrogen-bond geometries are provided in Tables S2–S5. The experimental powder XRD patterns of compounds **1**, **3**, and **4** (Figures S6–S8) closely match those simulated from the corresponding single-crystal models, confirming the phase purity of the synthesized materials. For compound **2**, the limited availability of crystalline material precluded the collection of powder XRD and optical data.

**Table 1:** Crystal Data, Data Collection, and Refinement Results for **1–4**, *T* = 295 K

	**1**	**2**	**3**	**4**
Crystal Data				
Chemical formula	(C_4_H_10_NO)_2_CsBiCl_6_	(C_4_H_10_NO)_2_CsSbCl_6_	(C_4_H_10_NO)_2_KBiCl_6_	(C_4_H_10_NO)_2_RbSbI_6_
*M*_r_	730.85	643.62	637.04	1144.88
Crystal system, space group	Monoclinic, *P*2_1_/*c*	Orthorhombic, *Pca*2_1_	Monoclinic, *I*2/*a*	Monoclinic, *C*2/*c*
*a*, *b*, *c* (Å)	16.519(5), 8.067(3), 15.730(5)	15.746(5), 8.055(3), 15.922(5)	15.194(5), 8.401(3), 31.349(8)	14.645(4), 10.034(4), 33.760(8)
β (°)	105.32(3)		92.78(3)	94.92(3)
*V* (Å^3^)	2021.7(12)	2019.5(12)	3997(2)	4943(3)
*Z*	4	4	8	8
μ (mm^–1^)	11.29	3.94	9.83	10.57
Crystal size (mm)	0.12 × 0.07 × 0.04	0.41 × 0.25 × 0.09	0.16 × 0.1 × 0.08	0.38 × 0.22 × 0.06
Data Collection				
*T*_min_, *T*_max_	0.440, 0.668	0.442, 0.746	0.869, 1.000	0.128, 0.555
Refl. measured, unique, observed [*I* > 2σ(*I*)]	13155, 4269, 2600	42221, 5233, 4724	9838, 4216, 3502	31587, 6152, 4820
*R*_int_	0.039	0.023	0.030	0.038
(sin θ/λ)_max_ (Å^–1^)	0.633	0.690	0.633	0.694
Refinement				
*R*[*F* ^2^ > 2σ(*F* ^2^)], *wR*(*F* ^2^), *S*	0.031, 0.053, 0.98	0.020, 0.038, 1.08	0.029, 0.048, 1.03	0.030, 0.056, 1.03
Data, parameters, restraints	4269, 185, 0	5233, 207, 37	4216, 183, 0	6152, 183, 0
Δρ_max_, Δρ_min_ (e Å^–3^)	0.81, −0.65	0.39, −0.49	0.57, −0.72	1.58, −0.87

Compound **1** crystallizes in the centrosymmetric monoclinic space group *P*2_1_/*c*. The asymmetric unit comprises two crystallographically independent Bi^3+^ ions located at inversion centers, one Cs^+^ ion, two morpholinium (MOR) cations, and six chloride anions (Figure 1a). The Cs^+^ ion is eight-coordinated by six chloride ligands and two oxygen atoms from two MOR cations, forming a distorted [CsCl_6_O_2_] dodecahedron. The Cs–Cl bond lengths range from 3.466(2) to 3.812(2) Å (Table S6). The Bi1-centered octahedron is corner-shared via chloride ligands with six [CsCl_6_O_2_] polyhedra, whereas the Bi2-centered octahedron is face-shared with two [CsCl_6_O_2_] units (Figure 1b). This connectivity generates a three-dimensional framework (Figure 1c). The morpholinium cations align along the *b* axis in an alternating fashion; symmetry-equivalent cations adopt the same orientation. Average Bi–Cl bond lengths are 2.712(3) Å (Bi1) and 2.705(3) Å (Bi2), with relatively small distortion indices (0.0030–0.0045) and moderate bond-angle variances (14.1–16.8 deg.^2^), indicating nearly regular BiCl_6_ octahedra. These values serve as a reference for comparison with the distortions observed in the other analogs (Table 2).

**Figure 1:**
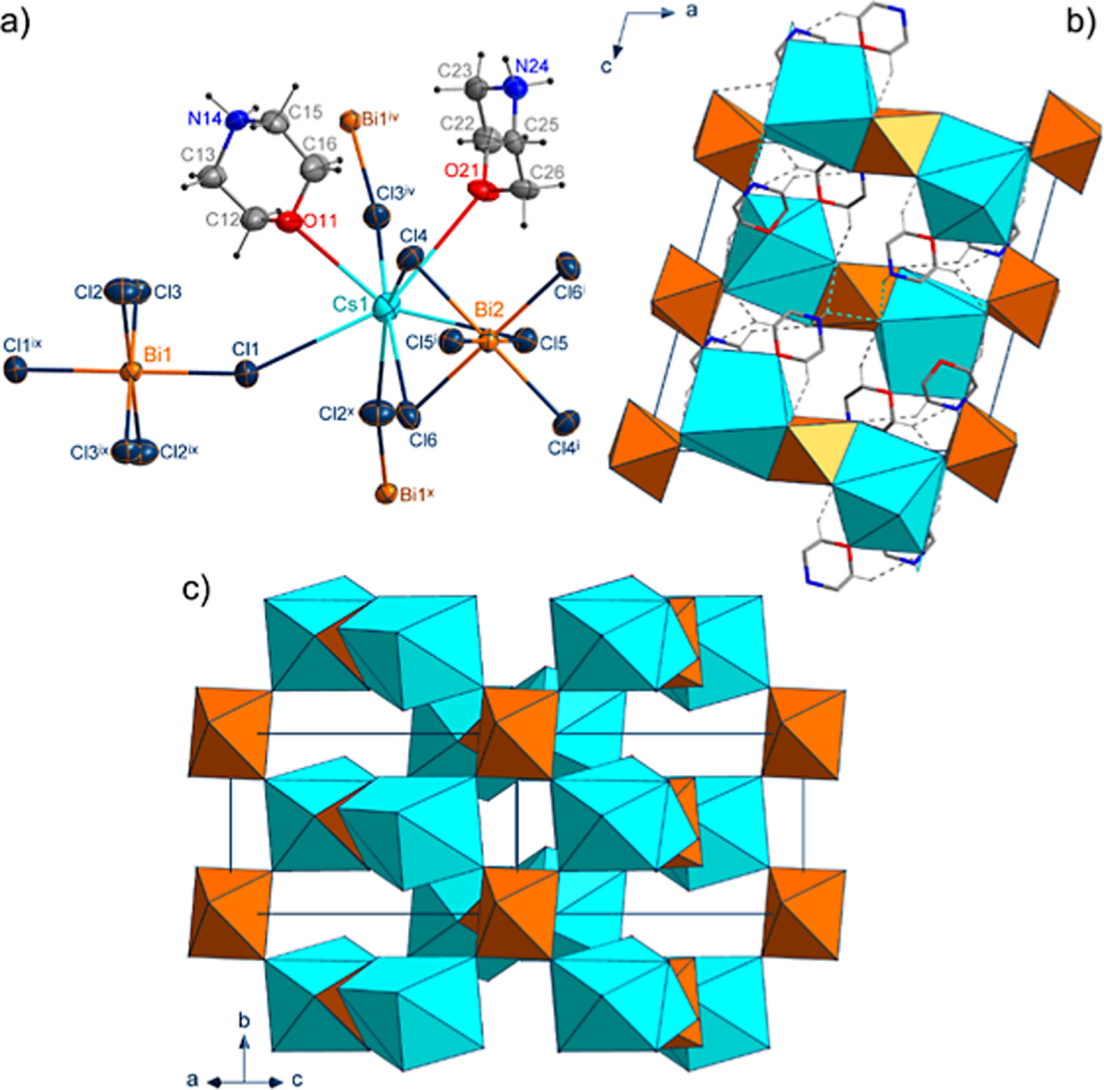
(a) The basic structural unit of compound **1** with atom-numbering scheme. (b) Crystal packing viewed along the [010] direction, showing the hydrogen-bond network (hydrogen atoms not involved in hydrogen bonds are omitted for clarity). (c) The view of the inorganic layer; morpholinium cations are omitted for clarity.

**Table 2:** Polyhedral Distortion Parameters of [M′X_6_] Octahedra in **1–4**

Compound	**1**		**2**	**3**		**4**	
	Bi1Cl_6_	Bi2Cl_6_	SbCl_6_	Bi1Cl_6_	Bi2Cl_6_	Sb1I_6_	Sb2I_6_
Average bond length, Å	2.712	2.705	2.663	2.711	2.710	3.037	3.029
Polyhedral volume, Å^3^	26.429	26.183	24.941	26.317	26.389	37.273	36.989
Distortion index	0.0030	0.0045	0.0237	0.0132	0.0079	0.0031	0.0178
Quadratic elongation	1.004	1.005	1.007	1.007	1.004	1.002	1.002
Bond angle variance, (°)^2^	14.068	16.836	21.098	23.457	12.704	5.998	5.901

The morpholinium cations establish an extensive hydrogen-bonding framework that stabilizes the three-dimensional structure. One cation donates three N–H···Cl interactions, including a bifurcated contact, while the other engages in four N–H···Cl bonds, one of which is trifurcated. These interactions, complemented by several C–H···Cl contacts (Table S2), result in a dense hydrogen-bonding network. The occurrence of bifurcated and trifurcated motifs highlights the ability of chloride to act as a versatile hydrogen-bond acceptor, reinforcing the linkage between the organic and inorganic structures.

Introducing Sb^3+^ in place of Bi^3+^ results in compound **2**, which adopts the orthorhombic noncentrosymmetric space group *Pca*2_1_. The asymmetric unit consists of one Sb^3+^ ion, one Cs^+^ ion, two MOR cations, and six chloride ligands, with all atoms occupying general positions (Figure 2a). One of the morpholinium cations is disordered over two orientations with refined occupancies of 0.75:0.25. The Cs^+^ ion is eight-coordinate, bound to six chloride ligands and two oxygen atoms from the organic cations, giving a [CsCl_6_O_2_] dodecahedron. The Sb-centered octahedron is connected to its surroundings by face-sharing with one [CsCl_6_O_2_] unit and by corner-sharing through three chloride atoms with three neighboring [CsCl_6_O_2_] polyhedra (Figure 2b). This arrangement produces a three-dimensional network (Figure 2c) that differs from that observed in compound **1**.

**Figure 2:**
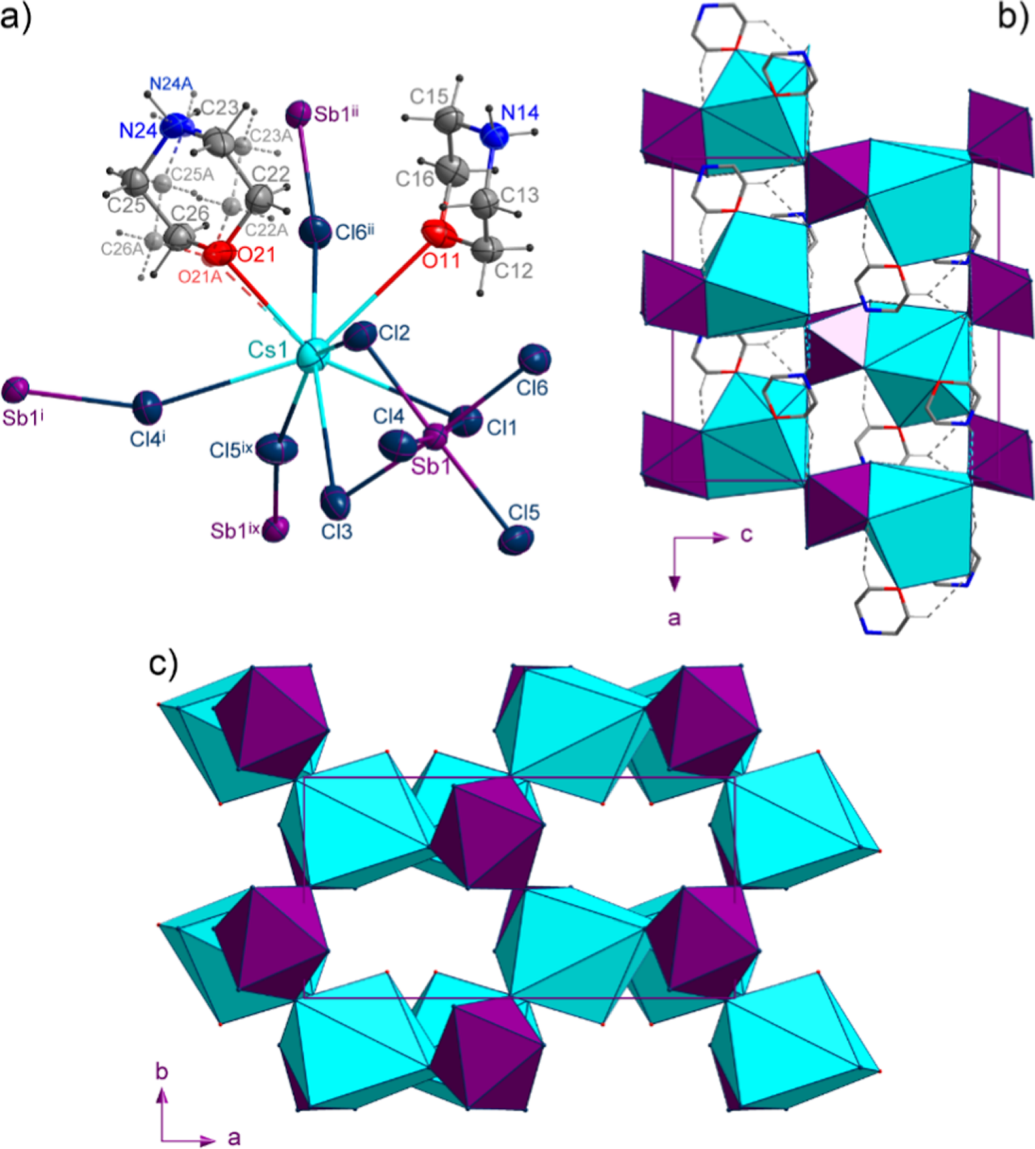
(a) The asymmetric unit of compound **2** with atom-numbering scheme. (b) Crystal packing viewed along the [010] direction, showing the hydrogen-bonding network. (c) The inorganic ring structure viewed along the [001] direction.

Sb–Cl bond lengths range from 2.594 to 2.784 Å, with an average of 2.663 Å (Table S6). The distortion index (0.0237) and bond-angle variance (21.1 deg.^2^) indicate pronounced octahedral distortions (Table 2), consistent with the stereochemical activity of the Sb^3+^ 5s^2^ lone pair and the noncentrosymmetric packing stabilized by morpholinium disorder.

The hydrogen-bonding system is extensive and involves both ordered and disordered cations. The first morpholinium cation forms up to five N–H···Cl interactions, whereas the disordered cation engages in shorter and more directional contacts (up to 171°) (Table S3). Numerous C–H···Cl interactions complement these contacts, together stabilizing the distorted SbCl_6_ framework. The absence of inversion symmetry reflects the off-centering tendency of Sb^3+^ in combination with partial ordering of the organic cations.

Low-temperature (100 K) measurements reveal that the previously disordered morpholinium cation becomes ordered upon cooling, indicating a freezing of dynamic disorder (Figure S9). However, this process does not induce any structural phase transition, as neither the framework connectivity nor the lattice parameters show significant changes between 100 and 295 K.

### 3.2 Correlations between Chemical Composition, Framework Dimensionality, and Structural Distortions

The structural diversity observed across the series arises from the systematic substitution of monovalent cations (Cs^+^, K^+^, Rb^+^), trivalent cations (Bi^3+^, Sb^3+^), and halides (Cl^–^, I^–^).

To ensure a consistent comparison, the discussion below combines the newly determined crystal structures of compounds **1** and **2** with re-evaluated diffraction data for compounds **3** and **4**, each refined under comparable experimental conditions.

This approach allows for a direct assessment of how cation size, electronic configuration, and halide identity collectively govern the dimensionality of the inorganic framework, the degree of octahedral distortion, and the nature and strength of hydrogen-bonding interactions within the hybrid lattice.

The K^+^ analog (compound **3**, Figure 3a) crystallizes in the monoclinic *I*2/*a* space group and is characterized by two independent Bi^3+^ sites, one K^+^ ion, two morpholinium cations, and six chloride ligands. The smaller size of K^+^ compared to Cs^+^ leads to a reduced coordination environment, forming a [KCl_4_O_2_] polyhedron in which four chloride and two oxygen atoms from morpholinium complete the coordination sphere. Along the [001] direction, BiCl_6_ octahedra alternate with [KCl_4_O_2_] units via edge-sharing, forming a one-dimensional chain surrounded by the organic matrix (Figure 3b). Average Bi–Cl bond lengths (2.7098–2.7111 Å, Table S8) and slightly higher distortion indices (0.0079–0.0132) than in compound **1** indicate increased octahedral distortion in the 1D structure. The hydrogen-bonding network connects the inorganic chains through three to four N–H···Cl and several C–H···Cl interactions (Table S4), resulting in fewer but stronger individual contacts than in compound **1**, consistent with the reduction from a 3D to a 1D framework.

**Figure 3:**
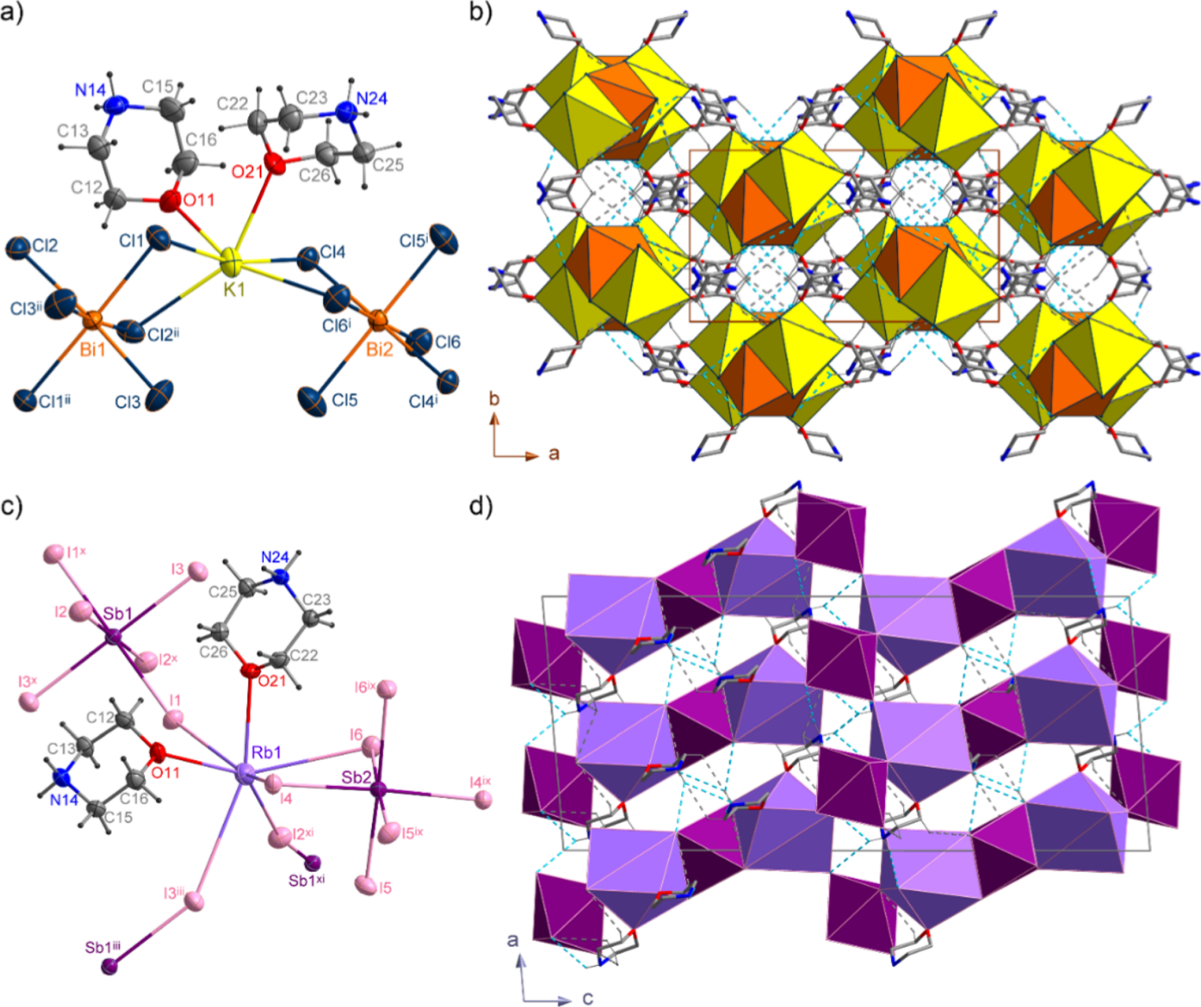
(a, c) The basic structural units of compounds **3** and **4**, respectively. (b) Crystal packing of compound **3** showing the one-dimensional chain along the *c*-axis with hydrogen bonds. (d) Crystal structure of compound **4** viewed along the [010] direction.

Replacing chloride with iodide and Cs^+^/K^+^ with Rb^+^ leads to compound **4**, which crystallizes in the monoclinic space group *C*2/*c*. The asymmetric unit (Figure 3c) contains one Rb^+^ ion, two independent Sb^3+^ centers, two morpholinium cations, and six iodide anions. The Rb^+^ ion is seven-coordinate, bonded to five iodide ligands and two oxygen atoms from morpholinium, forming a distorted [RbI_5_O_2_] polyhedron. The two distinct SbI_6_ octahedra exhibit corner- and edge-sharing connectivity, generating a three-dimensional network with ring-like motifs reminiscent of compounds **1** and **2** (Figure 3d). Average Sb–I bond lengths are 3.0359 and 3.0279 Å, with low distortion indices (0.0030–0.0175, Table S9) and minimal bond-angle variances (∼5.1 deg.^2^), indicating nearly regular SbI_6_ octahedra despite the elongated bonds. The substitution of Cl^–^ by the larger, more polarizable I^–^ results in bond elongation but reduced angular distortions, giving the most symmetric coordination geometry within the series.

The introduction of iodide also markedly modified the hydrogen-bonding pattern. Each morpholinium cation forms three to four N–H···I interactions, with H···I distances of approximately 3.0–3.2 Å and angles of 120–150°, which are longer and less linear than in the chloride analogs. Additional stabilization is provided by several C–H···I contacts (Table S5). Overall, the H-bonding network is weaker and more diffuse, consistent with the lower electronegativity and higher polarizability of iodide.

Furthermore, the intermolecular interactions in compounds **1**–**4** were examined using Hirshfeld surface analysis (Figures S10). The *d*_norm_ maps reveal pronounced red regions corresponding to the N–H···Cl/I and C–H···Cl/I contacts, highlighting the key role of hydrogen bonding in stabilizing all structures. The associated fingerprint plots confirm that H···Cl/I interactions constitute the largest contribution to the total Hirshfeld surface area in each compound, indicating that hydrogen bonding governs the overall crystal packing. The presence of these interactions is manifested as sharp, symmetric spikes in the lower region of the plots. The H···H contacts also contribute notably, and their proportion changes systematically with the structural dimensionality: higher for the one-dimensional chloride framework, and lower for the three-dimensional and iodide analogs. Finally, contributions from Cl···Cl/I···I interactions are observed, consistent with shorter halogen–halogen interactions in more tightly packed or more polarizable lattices.

Overall, the Hirshfeld surface analysis supports the X-ray diffraction findings, showing that hydrogen bonding dominates intermolecular organization, while the balance between H···H and X···X contacts correlates with changes in connectivity and halide identity. The quantitative contributions of the main intermolecular contacts (H···Cl/I, H···H, and Cl···Cl/I···I) are summarized in Table 3.

**Table 3:** Percentage Contributions of Selected Intermolecular Contacts to the Hirshfeld Surface Area for Studied Compounds

Compound	H···Cl/H···I(%)	H···H (%)	Cl···Cl/I···I
**1** (3D corner + face)	62.3	14.8	1.1
**2** (3D corner + face, polar)	68.3	16.6	0.3
**3** (1D edge chains)	66.7	19.3	1.0
**4** (3D corner + edge)	64.1	14.7	1.7

### 3.3 Electronic Structure and Charge-Carrier Properties of (MOR)_2_MBiCl_6_ and (MOR)_2_CsSbCl_6_ (M = Cs^+^, K^+^)

The electronic structures of hybrid halide compounds (MOR)_2_MBiCl_6_ (M = Cs^+^, K^+^) and (MOR)_2_CsSbCl_6_ were investigated using density functional theory (DFT) to elucidate the impact of alkali-metal cation substitution on their electronic structure and charge-transport properties. Calculated band structures and partial density of states (PDOS) are shown in Figures 4 and 5a. For all compounds, the valence band maximum (VBM) is dominated by σ-antibonding interactions between halide p and metal s orbitals, whereas the conduction band minimum (CBM) originates from hybridization of metal p and halide p states. The organic cation contributes mainly to deeper valence states and does not participate in the frontier electronic states responsible for the lowest-energy optical transitions.

**Figure 4:**
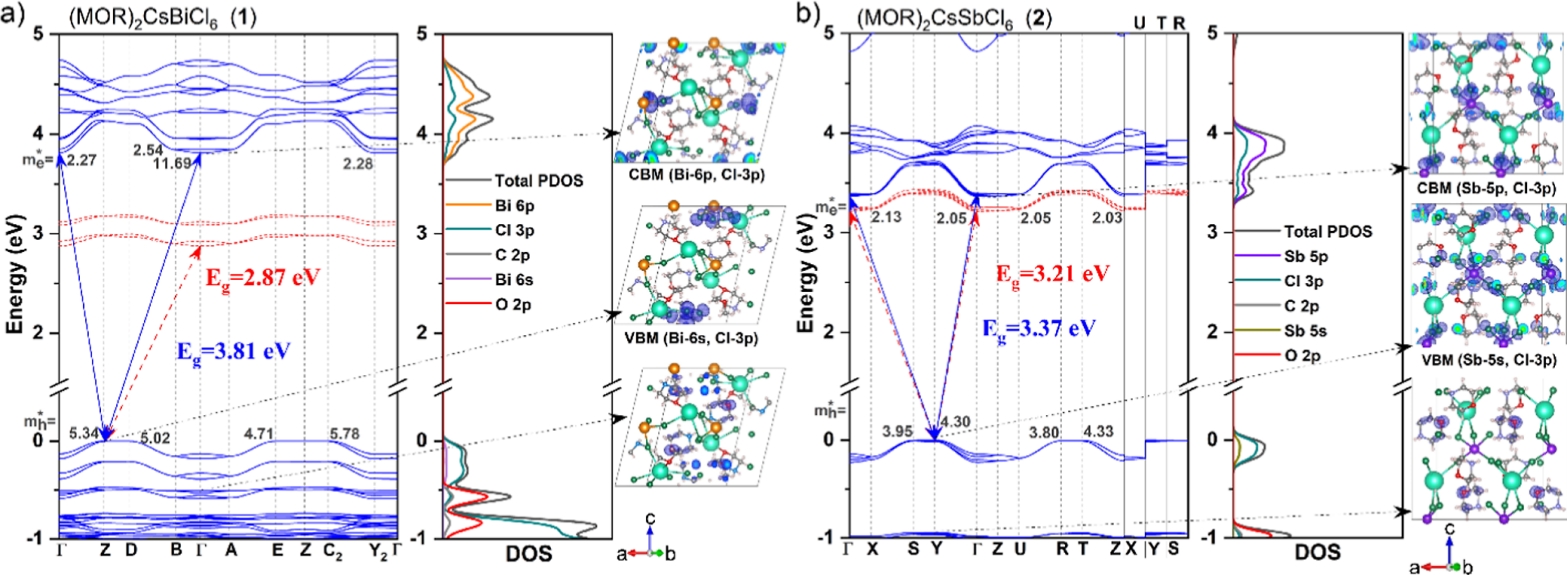
Scalar relativistic (blue solid line) and fully relativistic (red dashed line) band structure, together with scalar relativistic partial density of states (PDOS), for (a) compound **1** and (b) compound **2**.

**Figure 5:**
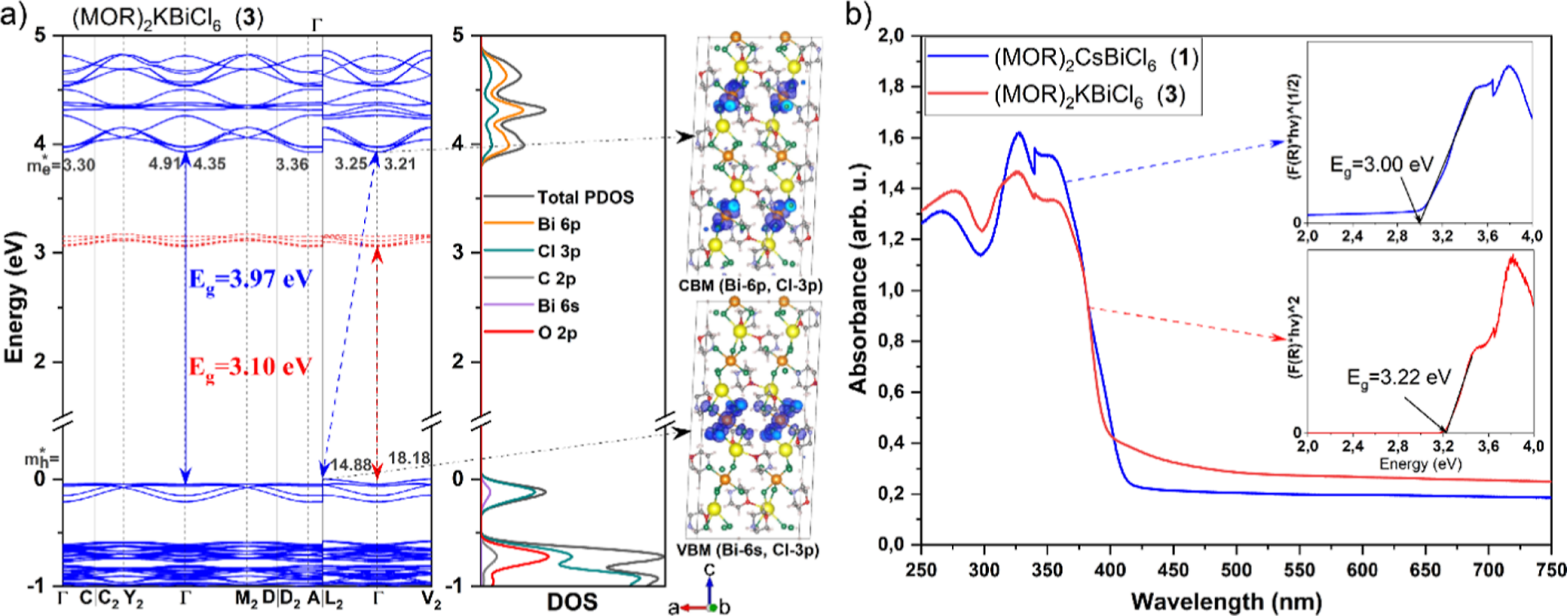
(a) Scalar relativistic (blue solid line) and fully relativistic (red dashed line) band structure, together with scalar relativistic PDOS, for compound **3**. (b) Experimental UV–vis absorption spectra of compounds **1** and **3**.

In compound **1**, the VBM is located at the *Z* point and the CBM at Γ, while in compound **2**, the corresponding band extrema occurs at the Y and Γ points, indicating indirect band gaps in both materials (Figure 4). In contrast, compound **3** exhibits a direct Γ-Γ transition (Figure 5a). The measured UV–vis absorption spectra and corresponding Tauc-plots are shown in Figure 5b. The experimental band gap of compound **1**, estimated using the Tauc method,^42^ is 3.00 eV and corresponds to an indirect transition, whereas compound **3** displays a direct band gap of 3.22 eV.

Scalar-relativistic GGA/PBE calculations significantly overestimate the band gaps, yielding values of 3.81 and 3.97 eV for **1** and **3**, respectively. Upon inclusion of spin–orbit coupling (SOC), the band gaps are reduced to 2.87 and 3.10 eV. In the antimony analog **2**, the SOC effect is less pronounced, lowering the calculated gap from 3.37 to 3.21 eV. These results demonstrate that SOC plays a decisive role in bismuth-containing compounds, where the heavy Bi^3+^ atom induces strong relativistic effects, leading to significant conduction-band splitting and a downward shift of the CBM. In contrast, the impact of SOC is weaker for Sb^3+^ due to its lower atomic mass. Notably, Cs-based compounds **1** and **2** retain an indirect band gap regardless of SOC inclusion.

The effective-mass calculations reveal pronounced anisotropy in charge-carrier transport, with electrons exhibiting higher mobility than holes. In compound **1**, the band dispersions along the Z–D, B−Γ, Z–C_2_, Y_2_–Γ, and E–Z, Γ–A paths are relatively flat, indicating strong localization of charge carriers along the *c* and *a* directions, as well as within the *ab* plane. In contrast, pronounced dispersions along the Γ–Z, D–B, E–A, and C_2_–Y_2_ paths indicate enhanced electron and hole mobility along the *b* direction. This anisotropic transport behavior correlates with shorter interoctahedral Bi–Cl···Cl–Bi distances along *b*, which facilitate charge transport via van der Waals interactions between adjacent BiCl_6_ octahedra. The calculated charge densities at the VBM and CBM indicate a clear spatial separation of holes and electrons, consistent with the formation of a direct Z-scheme heterojunction between the Bi(1)­Cl_6_ and Bi(2)­Cl_6_ octahedra (Figures 6 and S3). Electrons are predominantly localized on the Bi(1)­Cl_6_ units, whereas holes accumulate on the Bi(2)­Cl_6_ octahedra. The Cs^+^ cations act as insulating spacers between them, suppressing Fermi-level equilibration and preserving band offsets that promote charge separation. As a result, the spatially segregated charge distribution enhances the lifetime of photogenerated electron–hole pairs, indicating potential applicability in photocatalytic and photoelectrochemical systems. Band-structure analysis further reveals a quasi-one-dimensional charge-pathway along the *b* direction, with Bi(1)­Cl_6_ chains supporting electron transport and Bi(2)­Cl_6_ chains enabling hole conduction in the opposite direction. These pathways remain electronically decoupled due to the presence of intervening Cs^+^ ions. Comparable, albeit weaker, effects were previously reported for (MOR)_2_KInBr_6_ and (MOR)_2_KBiBr_6_,^43^ although they were not discussed in terms of spatial charge separation.

**Figure 6:**
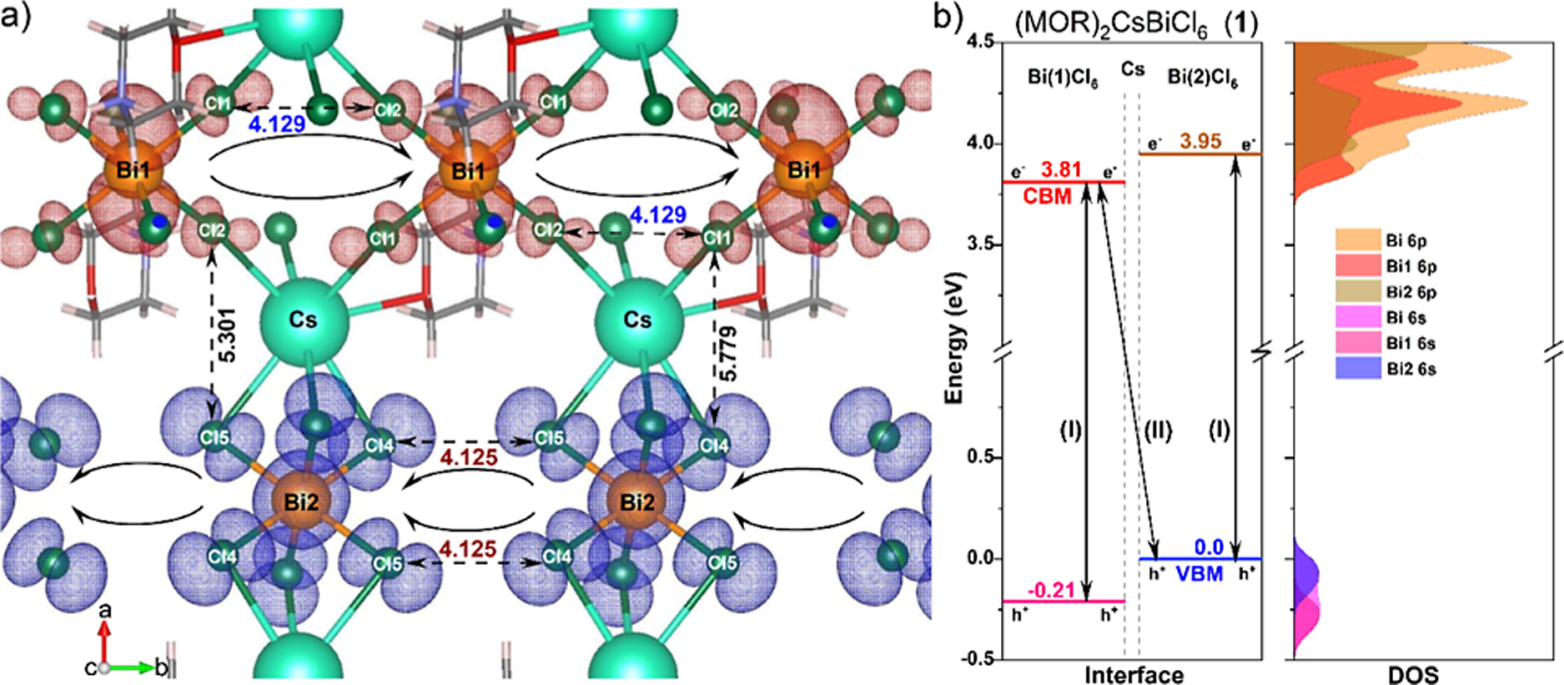
(a) Fragment of compound **1** showing selected geometric parameters (Å) and calculated electronic charge densities for the VBM (blue) and CBM (red). (b) Schematic representation of a Z-scheme heterojunction between crystallographically independent Bi(1)­Cl_6_ and Bi(2)­Cl_6_ units, together with scalar relativistic PDOS of Bi^3+^.

To experimentally confirm the electronic structure of **1**, photoluminescence (PL), photoluminescence excitation (PLE), and time-resolved emission (TRES) spectra were recorded under 305 nm excitation at 80 K. As shown in Figure 7, compound **1** exhibits intense blue photoluminescence in the 360–500 nm range. Notably, the emission profile differs from a Gaussian distribution, suggesting the presence of multiple PL bands. Time-resolved spectroscopy revealed two distinct emission bands with fundamentally different photophysical characters. The first emission band (P1) spans from 360 to 480 nm, with a maximum at 415 nm and Full Width at Half Maximum (fwhm) of 42 nm (Figure 7b,c). The short decay time of 9.2 ns (Figure 7d), as well as the excitation and emission spectra suggested that the band centered at 415 nm can be assigned to the characteristic Bi^3+^ emission originating from the ^3^P_0_ → ^1^S_0_ transition. Similar emission features have been widely reported for other low-dimensional hybrid Bi^3+^-based perovskites and halide materials^44,45^ (Figure 7d). Streak camera imaging clearly reveals that the second emission band (P2), centered at 440 nm with fwhm 43 nm, emerges with a measurable time delay relative to P1 (Figure 7b). The significantly longer radiative decay time, on the millisecond scale (1.22 ms), indicates a different recombination mechanism. In Bi-based halides, millisecond-scale luminescence may indeed arise from several mechanisms, including self-trapped excitons (STEs), defect/trap-mediated emission, phosphorescence/triplet-state recombination, or charge-transfer processes.^45–47^ However, several experimental and theoretical findings suggest that the P2 band cannot be readily explained by a conventional localized recombination mechanism. First, the Stokes shift and fwhm of the P2 emission band are too small to be considered a STE-type emission. The delayed onset of P2 relative to P1 observed in TRES measurements points to the involvement of an intermediate carrier-transfer process. Furthermore, the excitation spectra monitored at 415 and 440 nm differ in both peak position and spectral profile, indicating the presence of distinct emissive pathways. Importantly, DFT calculations reveal spatial separation of the frontier electronic states across the inequivalent Bi(1)­Cl_6_ and Bi(2)­Cl_6_ octahedra, providing a structural and electronic basis for charge-transfer recombination. This behavior is consistent with a Z-type heterojunction between the nonequivalent Bi(1)­Cl_6_ and Bi(2)­Cl_6_ octahedra, transition (II), as illustrated in Figure 6b, where electrons and holes are localized in separate units. The resulting spatial charge separation, further enhanced by insulating Cs^+^ spacers, delays electron–hole recombination, leading to prolonged decay times and the observed delayed emission.

**Figure 7:**
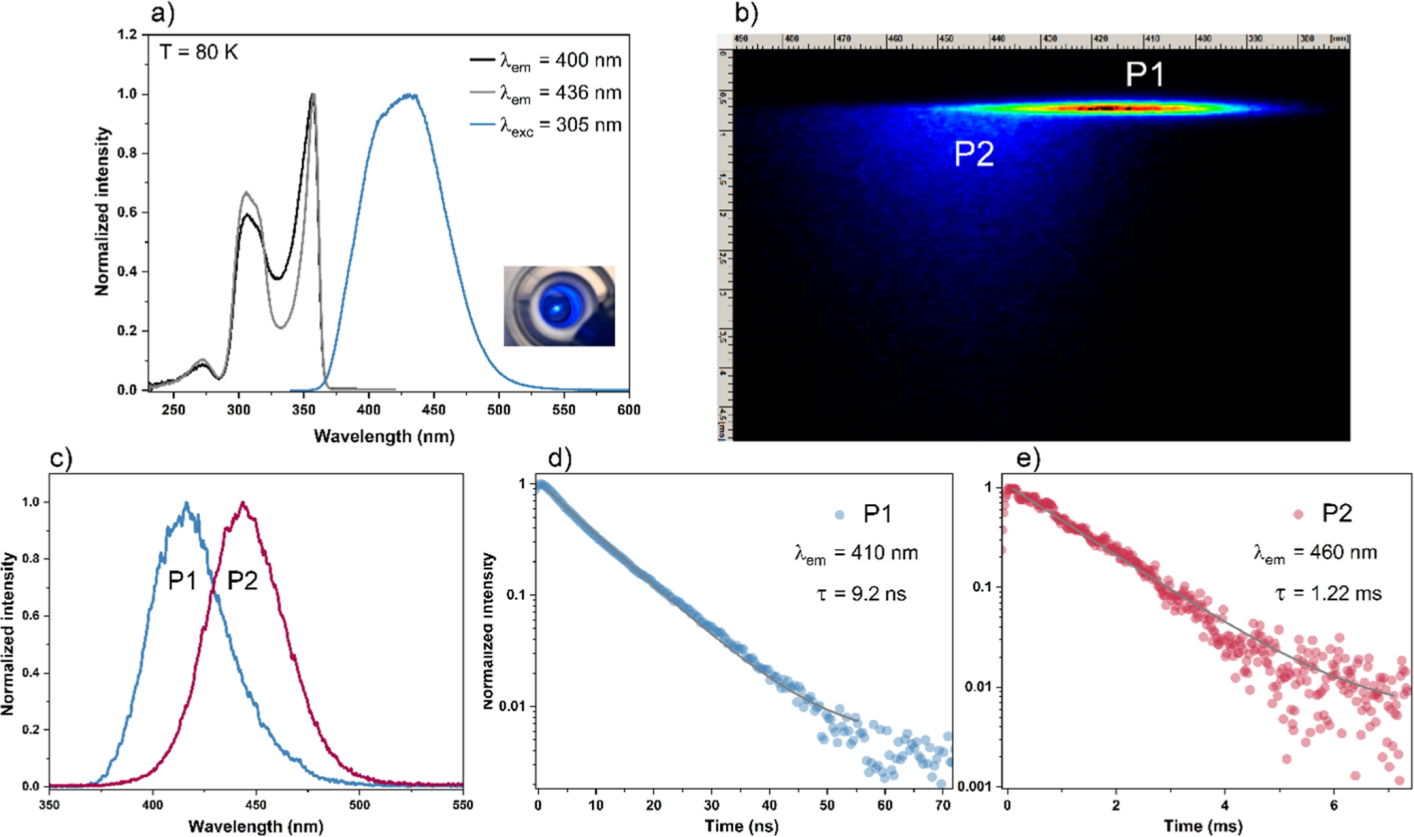
(a) Photoluminescence and photoluminescence excitation spectra of **1** recorded at 80 K; the inset shows a photograph of the sample exhibiting intense blue emission. (b) Time-resolved emission spectra recorded at 80 K under 305 nm excitation. (c) Spectral separation of two emission components, denoted as P1 and P2. (d,e) Luminescence decay curves of the P1 and P2 emission bands, respectively, derived from TRES.

Moreover, the temperature-dependent emission spectra recorded in the range of 80–310 K show that the position of the emission band maximum varies significantly with temperature. The areas identified by TRES as emission band maxima are marked in red and blue. As can be seen in Figure S11, the emission at 440 nm rapidly disappears with increasing temperature, whereas the emission at 415 nm is much more stable. A Z-type heterojunction between the nonequivalent Bi(1)­Cl_6_ and Bi(2)­Cl_6_ octahedra can be efficient at low temperatures, where phonon lattice vibrations are limited. However, an increase in temperature and, consequently, a greater contribution of vibrations causes the probability of this process to decrease, which is reflected in the spectra as a decrease in the intensity of the P2 emission. Considering both theoretical calculations and experimental results, we believe that the observed emission originates from both typical Bi transitions (P1 band) and is the result of a Z-type scheme, rather than STE emission, defect/trap-mediated emission, or phosphorescence/triplet-state recombination.

The anisotropy in charge-carrier transport is observed also in compound **2**, where charge-carrier pathways develop along the *b* direction. However, the presence of only one symmetry-inequivalent Sb site prevents the formation of effective spatial charge separation.

In contrast, the K-substituted compound **3** exhibits qualitatively different behavior. The valence-band maximum is nearly dispersionless, indicating strong hole localization and extremely low hole mobility, while electrons retain limited mobility along the *b* direction and within the *ab* plane. Structurally, the substitution of Cs^+^ by a smaller K^+^ cation markedly alters the interoctahedral connectivity, leading to reduced orbital overlap and suppressed charge transport. In compound **3**, the dimensionality of the metal-polyhedral framework is reduced, as BiCl_6_ octahedra alternate with [KCl_4_O_2_] units through edge-sharing, forming one-dimensional motifs surrounded by organic cations. This structural arrangement reduces van der Waals coupling between BiCl_6_ octahedra. The relatively large Cl···Cl separations (∼4.76 Å) suppress electronic overlap between adjacent Bi(1)­Cl_6_ units, leading to a quasi-zero-dimensional electronic character. Within the Bi(2)­Cl_6_ units, slightly shorter Cl···Cl contacts (∼4.55 Å) allow limited electron delocalization within the *ab* plane, giving rise to a quasi-two-dimensional electronic pathway; however, these distances remain too large to support efficient hole transport. Consequently, both bismuth environments possess nearly isoenergetic hole states, precluding effective spatial charge separation (Figure 8).

**Figure 8:**
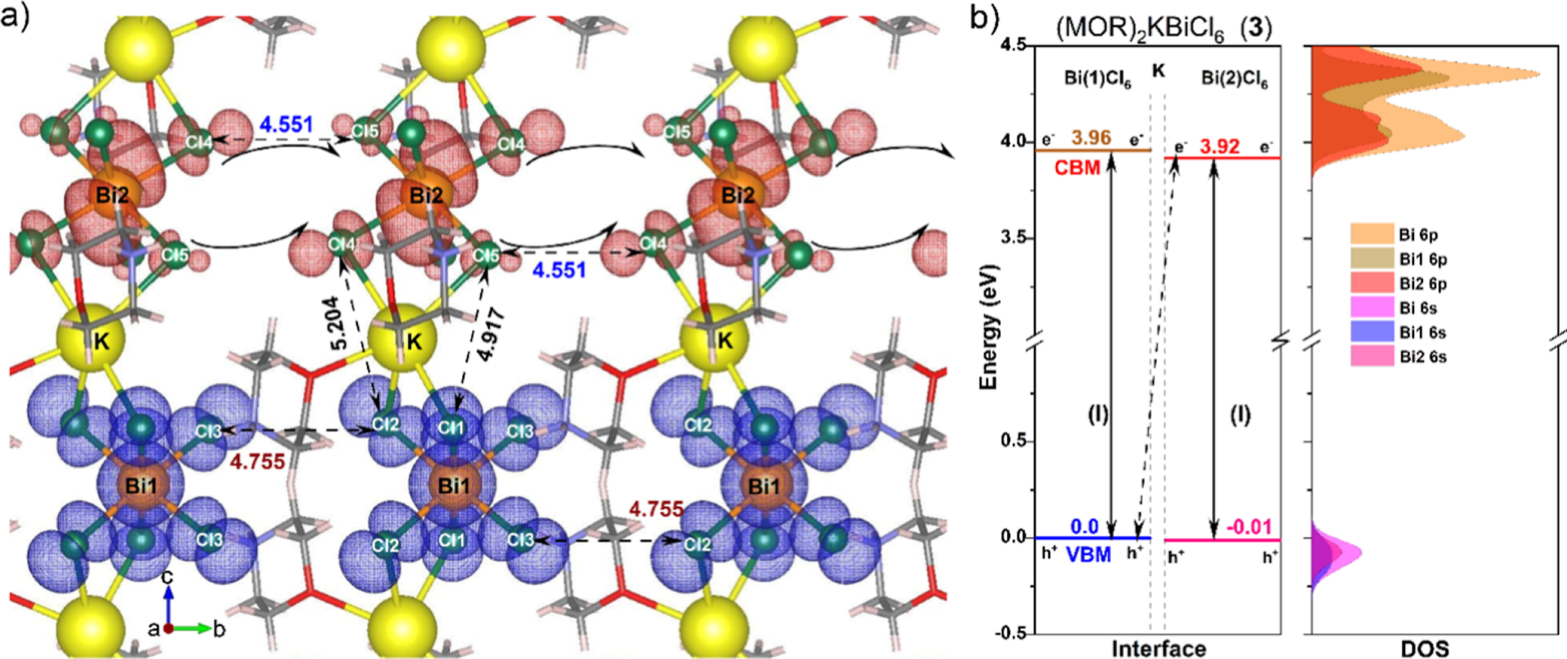
(a) Fragment of compound **3** with selected geometric parameters (Å) and calculated electronic charge densities for the VBM (blue) and CBM (red). (b) Schematic representation of the energetic diagrams between Bi(1)­Cl_6_ and Bi(2)­Cl_6_ units, together with scalar relativistic PDOS of Bi^3+^.

These observations highlight the important role of the alkali-metal cation in controlling both the structural dimensionality and electronic coupling in morpholinium-based Bi^3+^/Sb^3+^ halides. Cs^+^ cation stabilizes a framework, which promotes stronger Cl···Cl van der Waals interactions, enabling extended 1D charge-transport pathways along with efficient spatial charge separation. In contrast, substitution with the smaller K^+^ cation leads to a distinct structural arrangement characterized by increased interoctahedral separation, electronically isolated BiCl_6_ units, and strongly reduced carrier mobility. Thus, the choice of monovalent cation directly modulates interoctahedral distances, band dispersion, and ultimately the optoelectronic response of the material. Overall, (MOR)_2_CsBiCl_6_ (**1**) exhibits electronic features favorable for photoelectrochemical applications, whereas (MOR)_2_KBiCl_6_ (**3**) represents a structurally distinct and electronically localized analog.

## 4 Conclusions

Four compounds of the general formula (MOR)_2_MM′X_6_ were obtained, exhibiting remarkable diversity of architectures—ranging from one-dimensional chains to three-dimensional frameworks built from corner-, edge-, and face-sharing polyhedra. These results highlight that even within the same general formula of organic–inorganic hybrid compounds, subtle compositional and structural variations can lead to pronounced changes in framework dimensionality and local geometry, thereby influencing stability and electronic behavior.

Across **1**–**4**, the octahedral geometry is governed by the balance between halide size, trivalent cation character, and connectivity type. Compounds **2** and **3** exhibit the largest structural distortions, reflecting the stereochemically active Sb^3+^ lone pair and enhanced tilting associated with reduced dimensionality, respectively. In contrast, iodide incorporation favors more regular coordination environments by primarily affecting bond lengths rather than angular distortions.

Hydrogen-bonding interactions remain crucial for stabilizing all four frameworks, becoming progressively weaker and less directional upon substitution of chloride with iodide, as confirmed by Hirshfeld surface analysis. This highlights the cooperative role of hydrogen bonding and halide substitution on framework flexibility and stability.

The connectivity of BiCl_6_/SbX_6_ (X = Cl, I) octahedra and [MX_*n*_O_2_] polyhedra plays a decisive role in governing the electronic structure of hybrid halides. Experimental and theoretical studies have established that the optical band gap follows the sequence corner-sharing < edge-sharing < face-sharing, reflecting the decreasing orbital overlap between the metal ns^2^ lone pair and halide p orbitals. Within the series, distinct connectivity modes —corner-/face-sharing (**1** and **2**), edge-sharing chains (**3**), and mixed corner-/edge-sharing (**4**)—enable tuning of optoelectronic properties in morpholinium-based alkali bismuth and antimony halides.

Electronic structure calculations and experimental data reveal a clear structure–property relationship. Compounds **1** and **2** exhibit anisotropic charge transport, whereas **3** shows strongly localized electronic states due to its reduced dimensionality. In **1**, the presence of crystallographically inequivalent BiCl_6_ octahedra gives rise to spatial separation of band-edge charge densities and distinct photoluminescence pathways, suggesting potential relevance for optoelectronic applications.

Overall, these results establish that framework connectivity, cation choice, lone-pair activity, and halide substitution act cooperatively to govern both structure and electronic properties, providing rational design principles for tuning lead-free hybrid halide materials.

## Supplementary Material



## Data Availability

Meta-data are available at Zenodo DOI: 10.5281/zenodo.18231712.

## Notes

Estimated effective mass for the charge carriers for **1–3** (Table S1), schematic presentation of parabolic approximation near band extrema (Figure S1), scalar relativistic band structure for compound **1** without and with relaxation (Figure S2), scalar relativistic band structure of **1**, together with scalar relativistic PDOS of Bi^3+^ (Figures S3), Kubelka–Munk functions (Figures S4), scalar relativistic band structure and scalar relativistic partial density of states (PDOS) for (MOR)_2_RbSbI_6_ (Figure S5), crystallographic data (Tables S2–S9), powder X-ray diffraction (Figures S6–S8), asymmetric units (Figure S9), Hirshfeld surfaces and fingerprints (Figures S10), temperature-dependent emission spectra recorded at various temperatures of **1** (Figure S11) (PDF)
