# Supplementary material for: Connectivity-Driven Electronic Structure and Charge Separation in Morpholinium-Based Bi3+/Sb3+ Halides
Source: Inorg Chem. 2026 Jul 2;65(28):16482–94. doi: 10.1021/acs.inorgchem.6c02197 (PMC13390046; doi:10.1021/acs.inorgchem.6c02197)
Supplement: Supplementary file 1 [file ic6c02197_si_001.pdf]

Connectivity-Driven Electronic Structure and Charge Separation in Morpholinium-Based Bi^{3+}/Sb^{3+} Halides

Tamara J. Bednarchuk[a], Magdalena N. Rowińska[a],
Oleksandr Korolevych[a], Dagmara Stefańska[a] and Anna Gagor[a,*]

[a] *Institute of Low Temperature and Structural Research, Polish Academy of Science, Okólna 2, 50-422 Wrocław, Poland*

* Corresponding author: a.gagor@intibs.pl

Table S1 Estimated effective mass for the charge carriers in crystallographic directions (in m_e) for:

a) $(MOR)_2CsBiCl_6$ (**1**)

direction	Along *a* (Y_2–Γ)	Along *b* (Γ–Z)	Along *c* (Γ–B)
electron	∞	~2.3	~11.7
direction	Along *a* (C_2–Z)	Along -*b* (Γ–Z)	Along *c* (Z-D)
holes	∞	~5.3	∞

b) $(MOR)_2CsSbCl_6$ (**2**)

direction	Along *a* (Γ-X)	Along *b* (X–S)	Along *c* (X–U)
electron	∞	~2.10	∞
direction	Along *a* (T-R)	Along -*b* (S–X)	Along *c* (Γ-Z)
holes	∞	~4	∞

c) $(MOR)_2KBiCl_6$ (**3**)

direction	Along *a*	Along *b* (Γ–V_2)	Along *c* (Γ-Y_2)
electron	-	~3.2	-
direction	Along *a*	Along -*b* (V_2–Γ)	Along *c*
holes	∞	~18	∞

DFT calculations

Brillouin zone paths:

For **$(MOR)_2CsBiCl_6$** the Brillouin zone path was defined as Γ–Z–D–B–Γ–A–E–Z–C_2–Y_2–Γ, with the corresponding k-point coordinates: Z (0.0 0.5 0.0), D (0.0 0.5 0.5), B(0.0 0.0 0.5), A(-0.5 0.0 0.5), E (-0.5 0.5 0.5), C_2 (-0.5 0.5 0.0), and Y_2 (-0.5 0.0 0.0). For **$(MOR)_2CsSbCl_6$** the selected path was: Γ–X–S–Y–Γ–Z–U–R–T–Z|X–U|Y–T|S–R|, with k-point defined as: X (0.5 0.0 0.0), S (0.5 0.5 0.0), Y(0.0 0.5 0.0), Z(0.0 0.0 0.5), U (0.5 0.0 0.5), R (0.5 0.5 0.5), and T (0.0 0.5 0.5). For **$(MOR)_2KBiCl_6$**, the band structure was calculated along the path: Γ–C|C_2–Y_2–Γ–M_2–D|D_2–A–Γ|L_2–Γ–V_2, where the k-point coordinates are: C (0.268 0.268 0.0), C_2 (-0.268 0.732 0.0), Y_2(-0.5 0.5 0.0), M_2 (-0.5 0.5 0.5), D (-0.252 0.748 0.5), D_2 (0.252 0.252 0.5), A (0.0 0.0 0.5), L_2 (0.0 0.5 0.5), and V_2 (0.0 0.5 0.0). For **$(MOR)_2RbSbI_6$** (4) was depicted as Γ–C|C_2–Y_2–Γ–M_2–D|D_2–A–Γ|L_2–Γ–V_2 where C (0.368 0.368 0.0), C_2 (-0.368 0.632 0.0), Y_2(-0.5 0.5 0.0), M_2 (-0.5 0.5 0.5), D (-0.364 0.636 0.5), D_2 (0.364 0.364 0.5), A (0.0 0.0 0.5), L_2 (0.0 0.5 0.5), and V_2 (0.0 0.5 0.0).

The effective mass calculations.

The effective masses were determined from calculations performed without spin–orbit coupling (SOC), using the GGA/PBE functional. SOC was primarily included to improve the agreement between the calculated and experimental band-gap values, as discussed in the manuscript. However, the GGA/PBE calculations provide a more reliable basis for evaluating carrier effective masses, and the charge-density maps obtained from GGA/PBE calculations are sharper and less diffuse than those generated using GGA/PBE+SOC.

The effective masses were calculated using the standard relation

$$\frac{1}{m^*} = \frac{1}{\hbar^2}\frac{d^2E(k)}{dk^2},$$

where k is the wave vector along the transport direction and $E(k)$ represents the band eigenvalue energy.

In practice, the determination of effective masses involved more than a direct evaluation of the second derivative. The crucial step is converting the band eigenvalues to the correct units, according to the equation, which is not straightforward and depends on the output format of the computational software. Only then, selected regions near the band extrema were fitted using a parabolic approximation, as illustrated in Figure S1. The effective masses were then extracted from the quadratic fitting coefficient B_2 of (e.g., $E(k) = B_0 + B_1k + B_2k^2$) using the standard relation described in the manuscript and referenced earlier in the text.

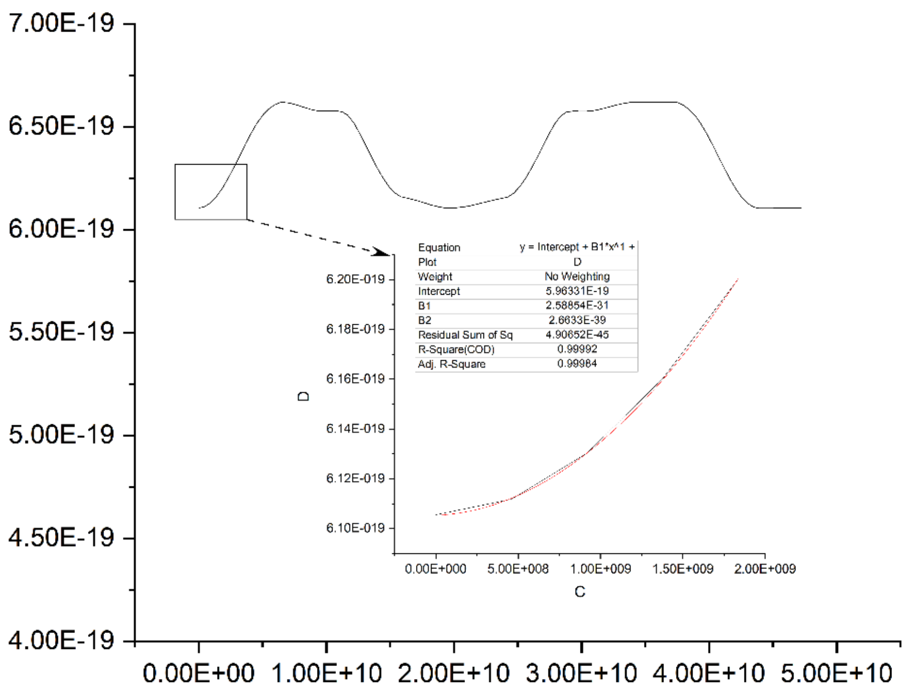

Figure S1. Schematic presentation of parabolic approximation near band extrema.

The methodology was additionally validated using independent software tools. In particular, the calculated effective masses were compared with the results obtained using Sumo (a Python toolkit) [https://zenodo.org/records/1338124]. The values obtained from Sumo were in good agreement with those produced by the selected methodology, confirming its reliability and consistency.

Band structure with and without geometry optimization.

We have performed geometry optimizations for **1** using the quasi-Newton Broyden-Fletcher-Goldfarb-Shanno (BFGS) algorithm scheme [2]. The following convergence criteria were set to: 10^{-5} Ry on total energy, 10^{-4} Ry for force convergence threshold, and 0.5 kbar for the cell gradient error. Marzari-Vanderbilt-DeVita-Payne [3] cold smearing was applied for electronic occupations, with a self-consistency convergence threshold of 10^{-6} Ry. The resulting structure has almost identical property, see Fig. S2.

The energy cutoffs.

The energy and charge density cutoffs for pseudopotentials were determined through a systematic trial calculation. Initially, they were set to 60 Ry for the wavefunctions and 400 Ry. Reducing the wavefunction cutoff to 40 Ry did not produce any significant change in the simulated outcome, while substantially decreasing computational cost. Additionally, these parameters are consistent with those reported for $Cs_3Bi_2I_9$ and $MA_3Bi_2I_9$ [4], and our own test calculation using the same setting reproduced reliable results.

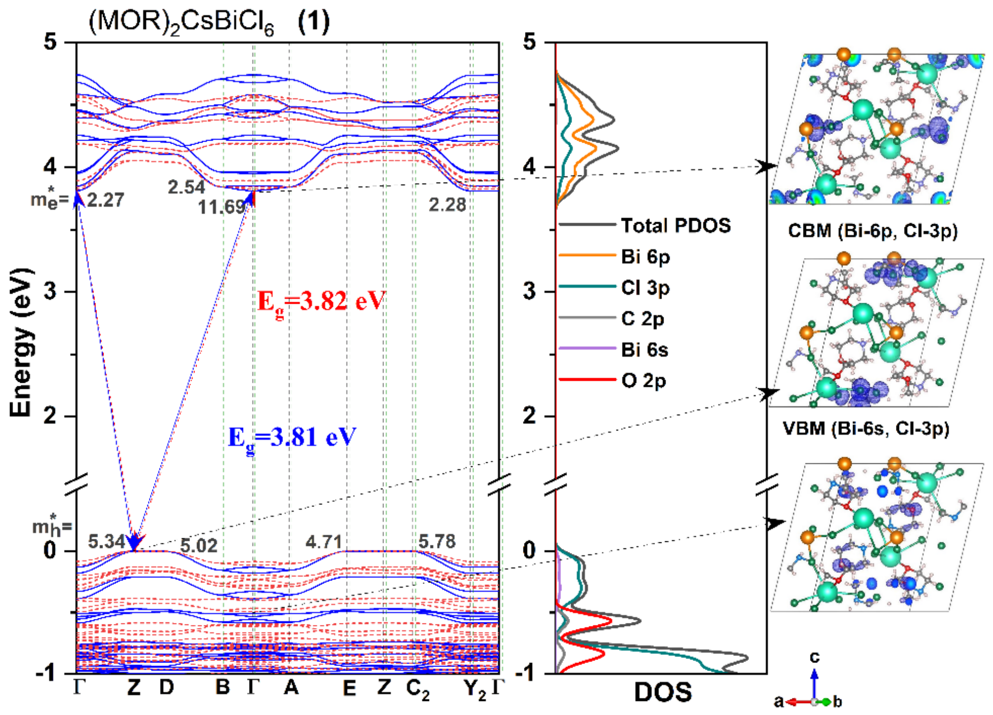

Figure S2. Scalar relativistic band structure without relaxation (blue solid line) and with relaxation (red dashed line), together with scalar relativistic PDOS, for compound **1**.

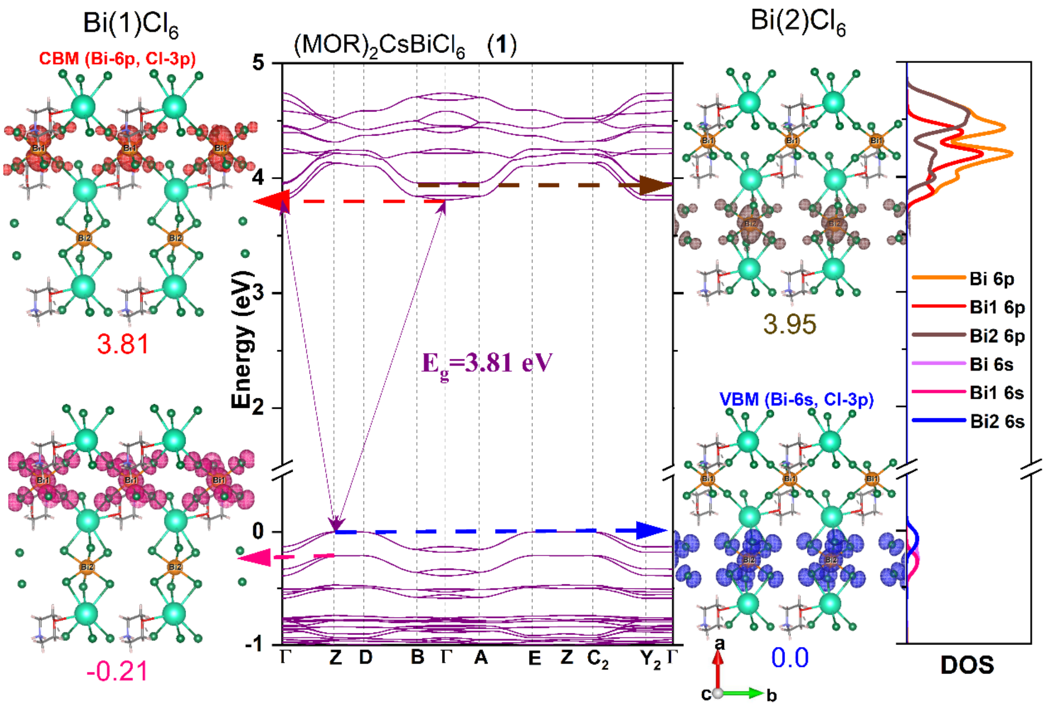

Figure S3. Scalar relativistic (violet solid line) band structure for **1**, together with scalar relativistic PDOS of Bi^{3+}. Fragment of compound showing calculated electronic charge densities CBM and VBM for the crystallographically independent $Bi(1)Cl_6$ and $Bi(2)Cl_6$ units.

Notes on Previously Reported DFT Data for the $(MOR)_2ABX_6$ Family

The compound $(MOR)_2KBiCl_6$, investigated by DFT in this work, was first mentioned within the $(MOR)_2ABX_6$ series (A = Na, K; B = In, Bi; X = Cl, Br) [5]. In that report, computational results were provided only for the bromide analogues $(MOR)_2KInBr_6$ and $(MOR)_2KBiBr_6$. The optical absorption spectra were reported for $(MOR)_2KBiCl_6$; however, the band-gap estimations were performed solely using the Kubelka–Munk transformation, without a Tauc analysis, which limits the precision of the optical transitions assigned to the observed absorption peaks.

Our transformed reflectance spectrum for $(MOR)_2KBiCl_6$ (Figure S4) indicates direct transition energies of 3.22 eV for peak (I) and 3.50 eV for peak (II). These features are reproduced in our GGA/PBE+SOC calculations, which yield values of 3.10 eV and 3.65 eV, respectively. Peak (I) corresponds to transitions from Bi s–Cl p states to hybridized Bi–Cl p orbitals, while peak (II) arises from deeper states associated with morpholine-derived orbitals mixed with Cl p states.

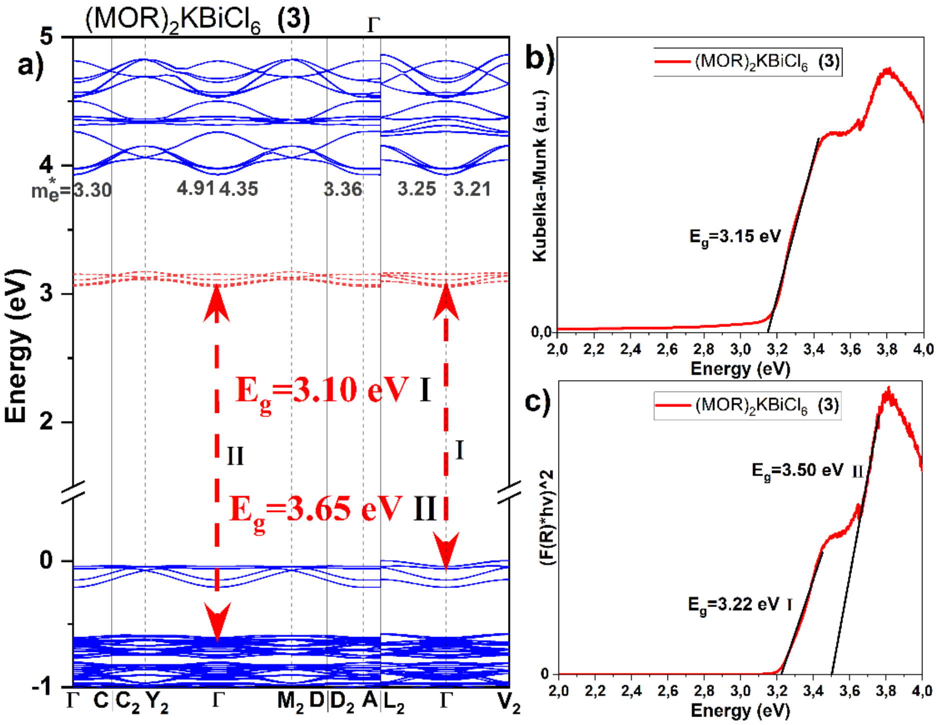

Figure S4. (a) Scalar relativistic (blue solid line) and fully relativistic (red dashed line) band structure **3**, (b) The Kubelka-Munk function (F(R∞) of **3**, (c) Transformed reflectance spectrum plot of **3** for direct transition.

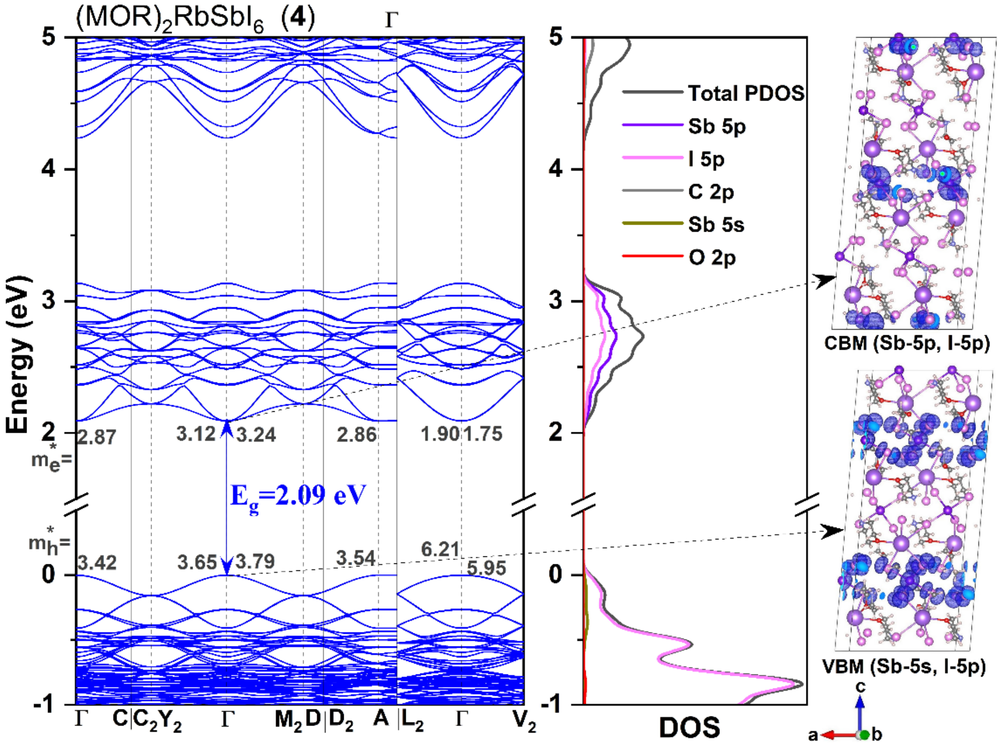

Figure S5. Scalar relativistic (blue solid line) band structure and scalar relativistic partial density of states (PDOS) for $(MOR)_2RbSbI_6$ (**4**).

Among the investigated compounds, only the iodine-containing hybrid structure exhibits a distinct direct band-gap transition located at the high-symmetry Γ point, with a calculated band-gap value of 2.09 eV. In contrast to the chlorine-containing analogue, the GGA/PBE approach provides a satisfactory description of the electronic structure, and the inclusion of spin–orbit coupling (SOC) was therefore not required.

The compositions of the valence-band maximum (VBM) and conduction-band minimum (CBM) remain essentially unchanged relative to **1**-**3** structures. The VBM is dominated by Sb-(s) and I-(p) orbitals, whereas the CBM is primarily derived from Sb-(p) and I-(p) states. Furthermore, the VBM and CBM are localized within different crystallographically independent octahedral units, namely $Sb(2)I_6$ and $Sb(1)I_6$ respectively.

$(MOR)_2RbSbI_6$ exhibits relatively high charge-carrier mobility. The holes display the highest mobility along the Γ–C and A–D_2 directions, corresponding predominantly to transport within the (ab) crystallographic plane. In contrast, the electrons are most mobile along the Γ–V_2 and Γ–L_2 directions, which are associated with charge transport along the *b* and *bc* crystallographic directions, respectively.

A comparison with previously published results [6] reveals a high degree of consistency. The

computational methodology employed in Ref. [6] was similar to that used in the present study, utilizing the Perdew–Burke–Ernzerhof functional revised for solids (PBEsol) together with norm-conserving pseudopotentials. The authors reported the same orbital contributions to the VBM and CBM, as well as their localization within different crystallographically independent octahedral units. However, no information regarding charge-carrier effective masses was provided.

The principal difference between the two studies concerns the nature of the band-gap transition. Whereas Ref. [6] reported an indirect band gap of 2.11 eV, our calculations predict a direct band-gap transition with a comparable band-gap value. This discrepancy may arise from the slightly different crystal metric and atomic parameters used for calculations in Ref. [6].

[1] Degen, T.; Sadki, M.; Bron, E.; König, U.; Nénert, G. *Powder Diffr*. **2014**, *29*, S13–S18.

[2] Fischer, T. H.; Almlof, J. *J. Phys. Chem*. **1992**, *96* (24), 9768–9774.

[3] Marzari, N.; Vanderbilt, D.; De Vita, A.; Payne, M. C. *Phys. Rev. Lett*. **1999**, *82*, 3296.

[4] Pazoki, M.; Johansson, M. B.; Zhu, H.; Broqvist, P.; Edvinsson, T.; Boschloo, G.; Johansson, E. M. J. *J. Phys. Chem. C* **2016**, *120* (51), 29039–29046.

[5] Li, R.; Zhou, Y.; Zhang, X.; Lin, J.; Chen, J.; Chen, C.; Pan, X.; Wang, P.; Chen, R.; Yin, J.; Mao, L. *Chem. Mater*. **2023**, *35* (21), 9362–9369.

[6] Fan, Q.; Xu, H.; Zhu, Z.-K.; Zhao, Z.; Rong, H.; Zhu, P.; Guo, W.; Tang, L.; Zhang, J.; Luo, J.; Sun, Z. 3D lead-free double perovskite via anchoring A-site cation for ultralow dose and stable X-Ray detection. *Adv. Funct. Mater*. **2025**, *35*, 2505546.

Table S2 Hydrogen-bond geometry (Å, º) for **1**

D—H···*A*	*D*—H	H···*A*	*D*···*A*	*D*—H···*A*
N14—H14*A*···Cl1^{ii}	0.89	2.45	3.276 (5)	154
N14—H14*B*···Cl3^{v}	0.89	2.60	3.291 (5)	135
N14—H14*B*···Cl2^{vi}	0.89	2.81	3.462 (5)	131
N24—H24*A*···Cl5^{iii}	0.89	2.83	3.464 (5)	129
N24—H24*A*···Cl4^{ii}	0.89	2.75	3.468 (5)	139
N24—H24*A*···Cl6^{ii}	0.89	2.88	3.424 (5)	121
N24—H24*B*···Cl5^{iv}	0.89	2.50	3.278 (5)	147
C12—H12*A*···Cl3^{i}	0.97	2.85	3.766 (8)	158
C12—H12*B*···Cl3	0.97	2.94	3.674 (7)	133
C12—H12*B*···Cl1	0.97	2.83	3.673 (8)	146
C15—H15*A*···Cl6^{ii}	0.97	2.92	3.637 (7)	131
C22—H22*A*···Cl3^{i}	0.97	2.88	3.675 (7)	140
C22—H22*A*···Cl2^{ii}	0.97	2.89	3.624 (7)	133
C23—H23*B*···Cl6^{ii}	0.97	2.89	3.566 (6)	128
C26—H26*B*···Cl5	0.97	2.75	3.562 (7)	142

Symmetry codes: (i) −*x*, *y*−1/2, −*z*+1/2; (ii) *x*, −*y*+1/2, *z*−1/2; (iii) −*x*+1, *y*+1/2, −*z*+1/2; (iv) *x*, −*y*−1/2, *z*−1/2; (v) −*x*, *y*+1/2, −*z*+1/2; (vi) *x*, −*y*+3/2, *z*−1/2.

Table S3 Hydrogen-bond geometry (Å, º) for **2**

D—H···*A*	*D*—H	H···*A*	*D*···*A*	*D*—H···*A*
N14—H14*A*···Cl2^{ii}	0.89	2.75	3.467 (3)	138
N14—H14*A*···Cl3^{ii}	0.89	2.85	3.421 (4)	123
N14—H14*A*···Cl4^{ii}	0.89	2.84	3.496 (4)	131
N14—H14*B*···Cl1^{i}	0.89	2.54	3.322 (3)	147
N14—H14*B*···Cl5^{i}	0.89	2.97	3.633 (4)	132
N24—H24*A*···Cl5^{iv}	0.89	2.75	3.410 (8)	132
N24—H24*A*···Cl6^{iv}	0.89	2.61	3.262 (16)	131

N24—H24*B*···Cl4^{iii}	0.89	2.46	3.285 (16)	154
N24*A*—H24*C*···Cl4^{iii}	0.89	2.49	3.19 (5)	136
N24*A*—H24*D*···Cl6^{iv}	0.89	2.51	3.39 (5)	171
C12—H12*A*···Cl3^{i}	0.97	2.97	3.897 (5)	160
C12—H12*B*···Cl1	0.97	2.76	3.609 (4)	146
C12—H12*B*···Cl6	0.97	2.97	3.670 (4)	130
C15—H15*B*···Cl3^{ii}	0.97	2.95	3.606 (5)	126
C16—H16*A*···Cl5^{iii}	0.97	2.95	3.698 (4)	135
C16—H16*A*···Cl6^{iii}	0.97	2.91	3.652 (5)	134
C23*A*—H23*C*···Cl3^{ii}	0.97	2.87	3.57 (2)	130
C26—H26*A*···Cl4^{v}	0.97	2.77	3.618 (6)	146
C26—H26*B*···Cl6^{iii}	0.97	2.85	3.759 (7)	156
C26*A*—H26*C*···Cl5^{v}	0.97	2.77	3.462 (19)	129
C26*A*—H26*D*···Cl4^{v}	0.97	2.77	3.646 (19)	151

Symmetry codes: (i) $x-1/2$, $-y+1$, z; (ii) $x-1/2$, $-y+2$, z; (iii) $-x+1/2$, y, $z+1/2$; (iv) $-x+1/2$, $y+1$, $z+1/2$; (v) $-x+1$, $-y+2$, $z+1/2$.

Table S4 Hydrogen-bond geometry (Å, º) for **3.**

D—H···*A*	*D*—H	H···*A*	*D*···*A*	*D*—H···*A*
N14—H14*A*···Cl2^{i}	0.89	2.62	3.292 (4)	133
N14—H14*A*···Cl3^{i}	0.89	2.79	3.459 (4)	133
N14—H14*B*···Cl1^{iv}	0.89	2.42	3.271 (4)	159
N24—H24*A*···Cl4^{i}	0.89	2.36	3.233 (4)	168
N24—H24*B*···Cl5^{ii}	0.89	2.91	3.576 (4)	133
N24—H24*B*···Cl6^{ii}	0.89	2.70	3.380 (4)	134
N24—H24*B*···Cl4^{iii}	0.89	2.87	3.450 (4)	125
C12—H12*A*···Cl3^{iv}	0.97	2.88	3.781 (6)	156
C12—H12*B*···Cl2	0.97	2.78	3.512 (5)	133
C25—H25*B*···Cl5	0.97	2.98	3.799 (5)	143

C26—H26*A*···Cl4	0.97	2.88	3.738 (5)	148
C26—H26*B*···Cl6^{i}	0.97	2.89	3.778 (5)	153

Symmetry codes: (i) *x*+1/2, −*y*+1, *z*; (ii) *x*+1/2, −*y*, *z*; (iii) −*x*+1, *y*−1/2, −*z*+1/2; (iv) *x*, *y*+1, *z*.

Table S5 Hydrogen-bond geometry (Å, º) for **4.**

D—H···*A*	*D*—H	H···*A*	*D*···*A*	*D*—H···*A*
N14—H14A···I1^{i}	0.89	3.13	3.784 (5)	132
N14—H14A···I2^{ii}	0.89	3.13	3.756 (4)	129
N14—H14B···I3^{iii}	0.89	2.91	3.715 (5)	152
N24—H24A···I4^{vii}	0.89	3.04	3.708 (4)	133
N24—H24A···I5^{viii}	0.89	3.23	3.923 (4)	137
N24—H24B···I4^{vi}	0.89	3.19	3.862 (5)	134
N24—H24B···I6^{v}	0.89	3.03	3.751 (4)	140
C12—H12A···I1	0.97	3.18	3.890 (6)	132
C15—H15A···I1^{iii}	0.97	3.21	3.868 (5)	127
C15—H15A···I2^{iv}	0.97	3.28	3.886 (5)	122
C15—H15B···I1^{i}	0.97	3.23	3.879 (7)	126
C16—H16A···I5^{v}	0.97	3.29	3.987 (6)	130
C22—H22A···I4	0.97	3.25	3.945 (5)	130
C22—H22A···I6	0.97	3.18	3.896 (5)	132
C23—H23A···I4^{vi}	0.97	3.24	3.837 (6)	122

Symmetry codes: (i) −*x*+1/2, *y*+1/2, −*z*+1/2; (ii) *x*, *y*+1, *z*; (iii) −*x*+1, *y*+1, −*z*+1/2; (iv) *x*−1/2, *y*+1/2, *z*; (v) −*x*+1/2, −*y*+3/2, −*z*; (vi) −*x*, −*y*+2, −*z*; (vii) *x*−1/2, *y*−1/2, *z*; (viii) *x*, *y*−1, *z*.

Table S6 Selected Bi–Cl and Cs–Cl bond lengths (Å) in compound **1**.

Bi1—Cl3	2.700 (2)	Bi2—Cl4	2.7002 (17)
Bi1—Cl3^{i}	2.700 (2)	Bi2—Cl6^{ii}	2.6900 (17)
Bi1—Cl1	2.7241 (16)	Bi2—Cl6	2.6900 (17)
Bi1—Cl1^{i}	2.7241 (16)	Cs1—Cl5	3.5014 (18)
Bi1—Cl2^{i}	2.7104 (16)	Cs1—Cl3^{iii}	3.466 (2)

Bi1—Cl2	2.7104 (17)	Cs1—Cl4	3.4776 (18)
Bi2—Cl5	2.7226 (16)	Cs1—Cl1	3.6655 (18)
Bi2—Cl5^{ii}	2.7226 (16)	Cs1—Cl2^{iv}	3.6296 (18)
Bi2—Cl4^{ii}	2.7002 (17)	Cs1—Cl6	3.812 (2)

Symmetry codes: (i) −*x*, −*y*+1, −*z*+1; (ii) −*x*+1, −*y*, −*z*+1.

Table S7 Selected Sb–Cl and Cs–Cl bond lengths (Å) in compound **2**.

Sb1—Cl1	2.5943 (12)	Cs1—Cl1	3.5155 (14)
Sb1—Cl2	2.5998 (11)	Cs1—Cl2	3.4326 (14)
Sb1—Cl3	2.6055 (12)	Cs1—Cl3	3.9373 (16)
Sb1—Cl4	2.7844 (13)	Cs1—Cl4^{i}	3.6074 (12)
Sb1—Cl5	2.7224 (12)	Cs1—Cl5^{ii}	3.5269 (13)
Sb1—Cl6	2.6707 (12)	Cs1—Cl6^{iii}	3.4407 (13)

Symmetry codes: (i) −*x*+1, −*y*+2, *z*+1/2; (ii) −*x*+1, −*y*+1, *z*+1/2; (iii) −*x*+1/2, *y*, *z*+1/2.

Table S8 Selected Bi–Cl and K–Cl bond lengths (Å) in compound **3**.

Bi1—Cl1^{i}	2.7362 (15)	Bi2—Cl5^{ii}	2.7074 (13)
Bi1—Cl1	2.7363 (15)	Bi2—Cl5	2.7075 (13)
Bi1—Cl2	2.7396 (13)	Bi2—Cl6	2.6799 (15)
Bi1—Cl2^{i}	2.7395 (13)	Bi2—Cl6^{ii}	2.6800 (15)
Bi1—Cl3	2.6574 (13)	Bi2—Cl4^{ii}	2.7418 (12)
Bi1—Cl3^{i}	2.6574 (13)	Bi2—Cl4	2.7418 (12)
Cl1—K1	3.0768 (17)	Cl5—K1	3.1893 (17)
Cl2—K1	3.1688 (19)	K1—Cl4	3.1044 (16)

Symmetry codes: (i) −*x*+1/2, *y*, −*z*; (ii) −*x*+1/2, −*y*+1/2, −*z*+1/2.

Table S9 Selected Sb–I and Rb–I bond lengths (Å) in compound **4**.

Sb1—I1^{i}	3.0511 (9)	Sb2—I4	3.0215 (10)
Sb1—I1	3.0511 (9)	Sb2—I4^{iv}	3.0215 (10)
Sb1—I2^{i}	3.0324 (10)	Sb2—I5^{iv}	2.9560 (9)
Sb1—I2	3.0325 (10)	Sb2—I5	2.9561 (9)
Sb1—I3^{i}	3.0282 (8)	Sb2—I6	3.1106 (9)

Sb1—I3	3.0281 (8)	Sb2—I6^{iv}	3.1106 (9)
I1—Rb1	3.7495 (10)	I4—Rb1	3.6264 (11)
I2—Rb1^{ii}	3.9333 (13)	I6—Rb1	3.6630 (13)
I3—Rb1^{iii}	3.8892 (14)		

Symmetry codes: (i) −*x*+1/2, −*y*+5/2, −*z*; (ii) *x*−1/2, *y*+1/2, *z*; (iii) *x*, *y*+1, *z*; (iv) −*x*+1, *y*, −*z*+1/2.

XRD Analysis.

The experimental diffraction patterns were compared with theoretical patterns obtained from SC-XRD using the Rietveld method with the use of HighScore Plus [1] (Figs. S6–S8).

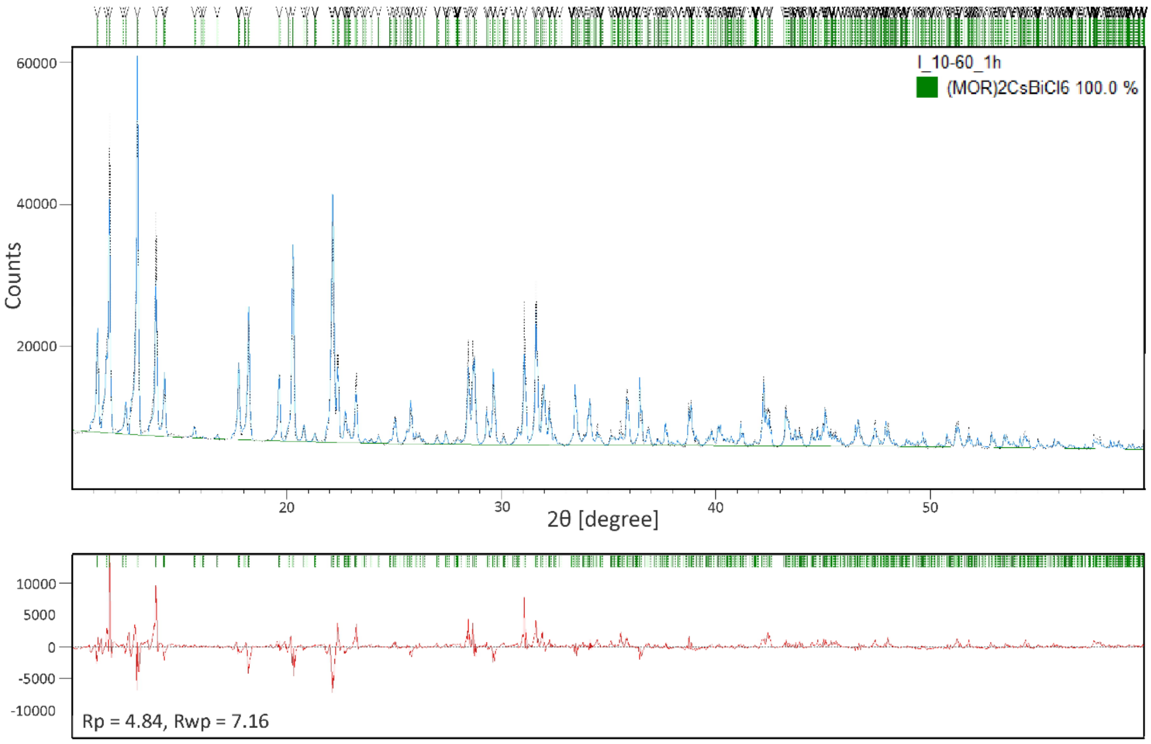

Figure S6. Rietveld refinement plot of compound **1**. Black circles represent the experimental PXRD pattern, the blue line corresponds to the calculated profile, the red line shows the difference curve, and vertical green tick marks indicate the Bragg reflection positions.

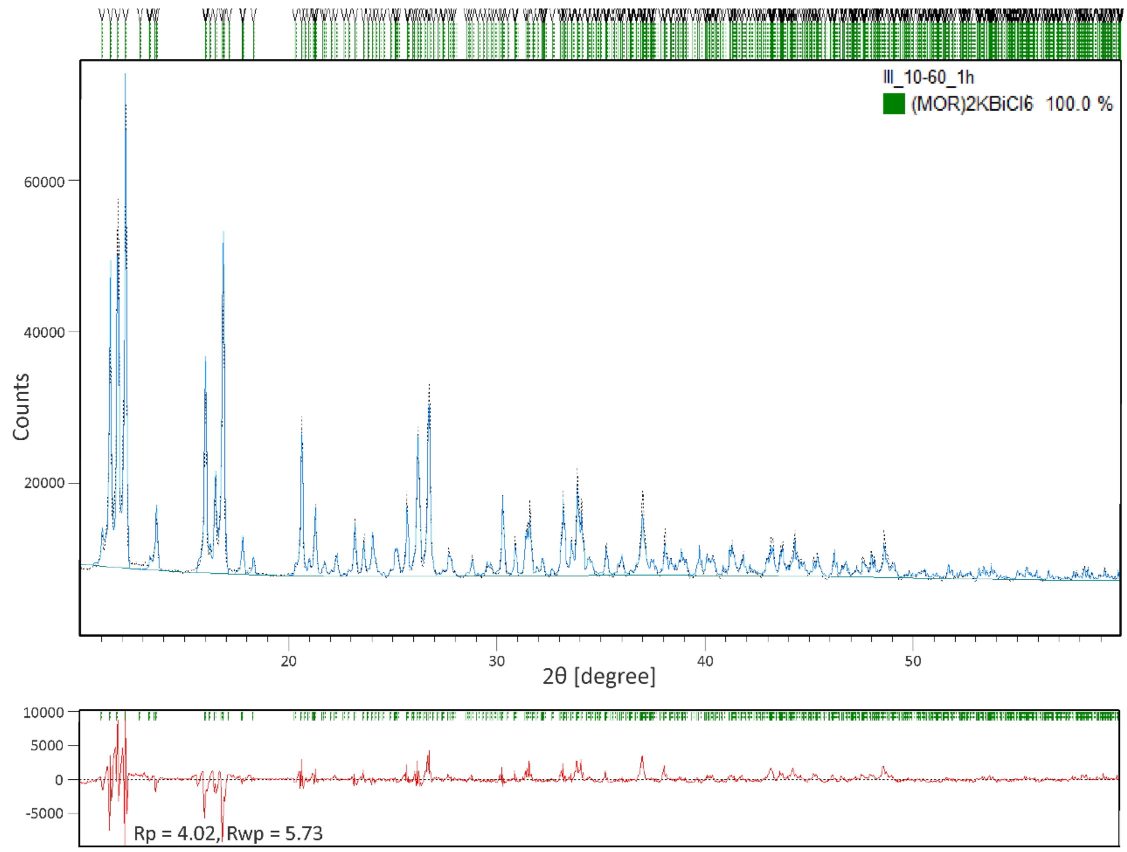

Figure S7. Rietveld refinement plot of compound **3**. Black circles represent the experimental PXRD pattern, the blue line corresponds to the calculated profile, the red line shows the difference curve, and vertical green tick marks indicate the Bragg reflection positions.

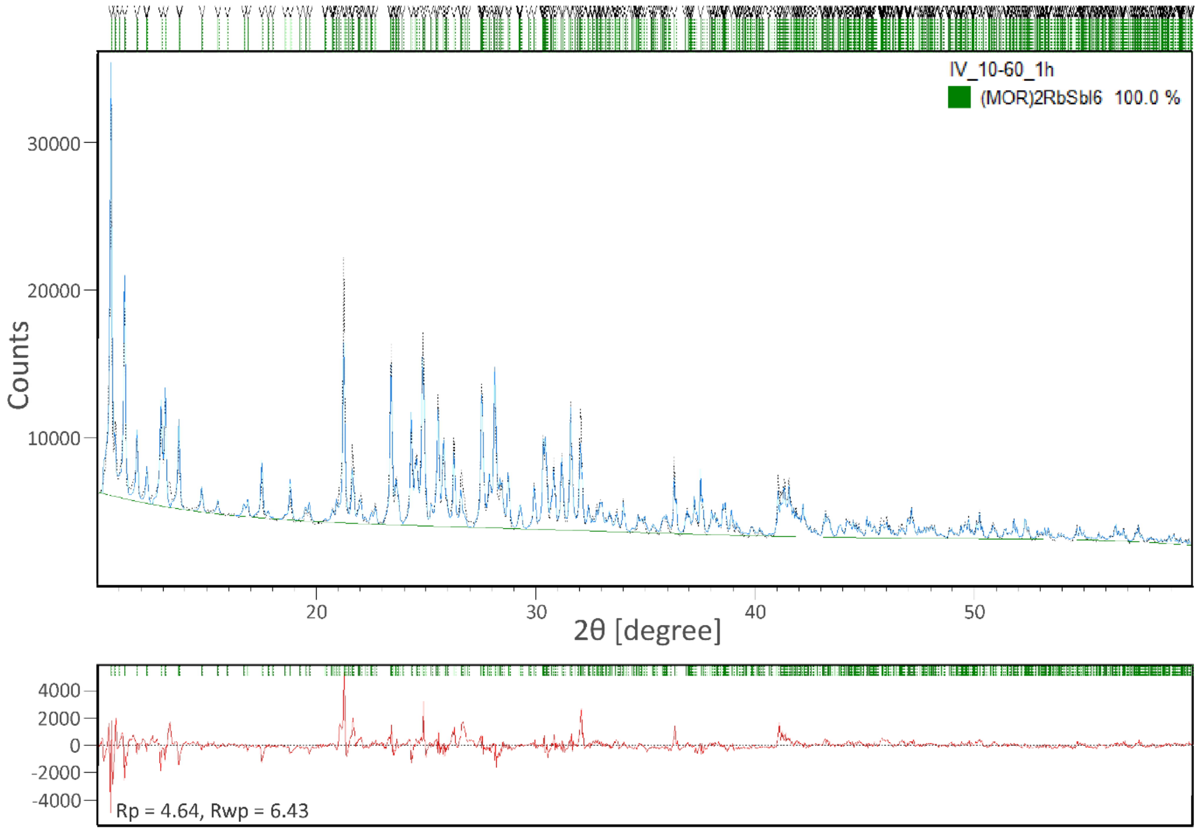

Figure S8. Rietveld refinement plot of compound **4**. Black circles represent the experimental PXRD pattern, the blue line corresponds to the calculated profile, the red line shows the difference curve, and vertical green tick marks indicate the Bragg reflection positions.

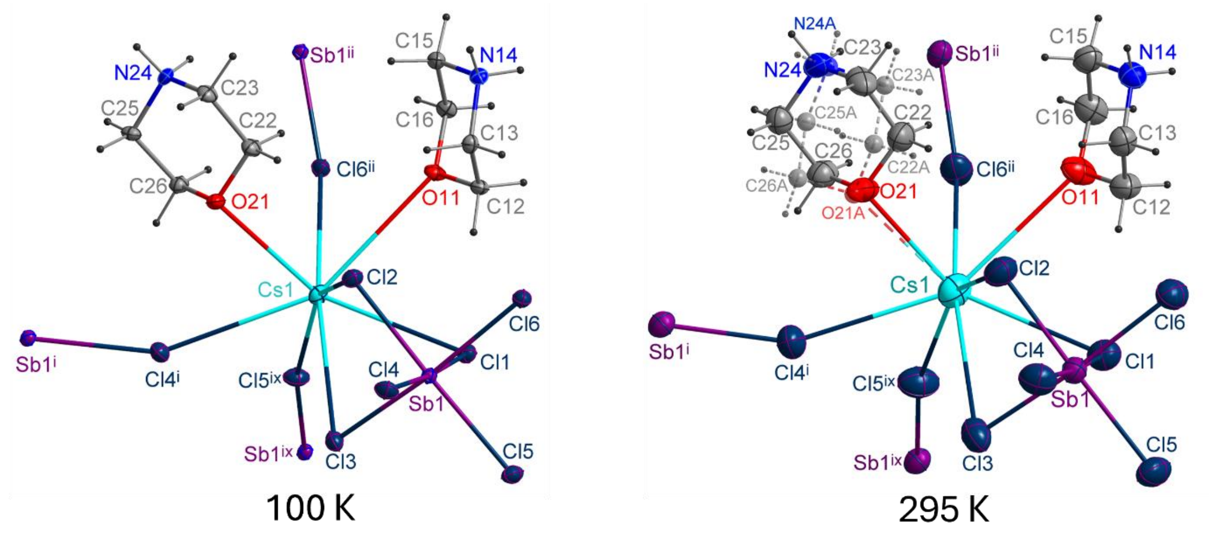

Figure S9. The asymmetric unit of compound **2** with atom-numbering scheme at 100 K and 295 K. Symmetry codes: (i) −*x*+1, −*y*+2, *z*+1/2; (ii) −*x*+1, −*y*+1, *z*+1/2; (ix) 1−*x*, 1−*y*, 1/2+*z*.

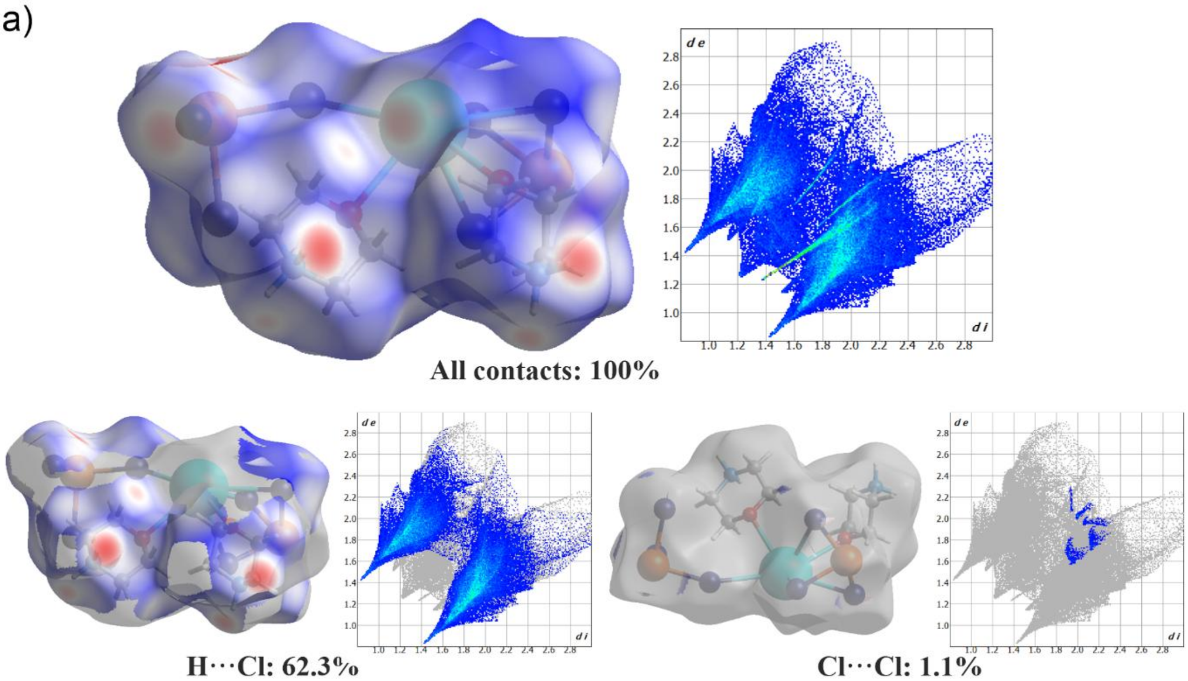

b)

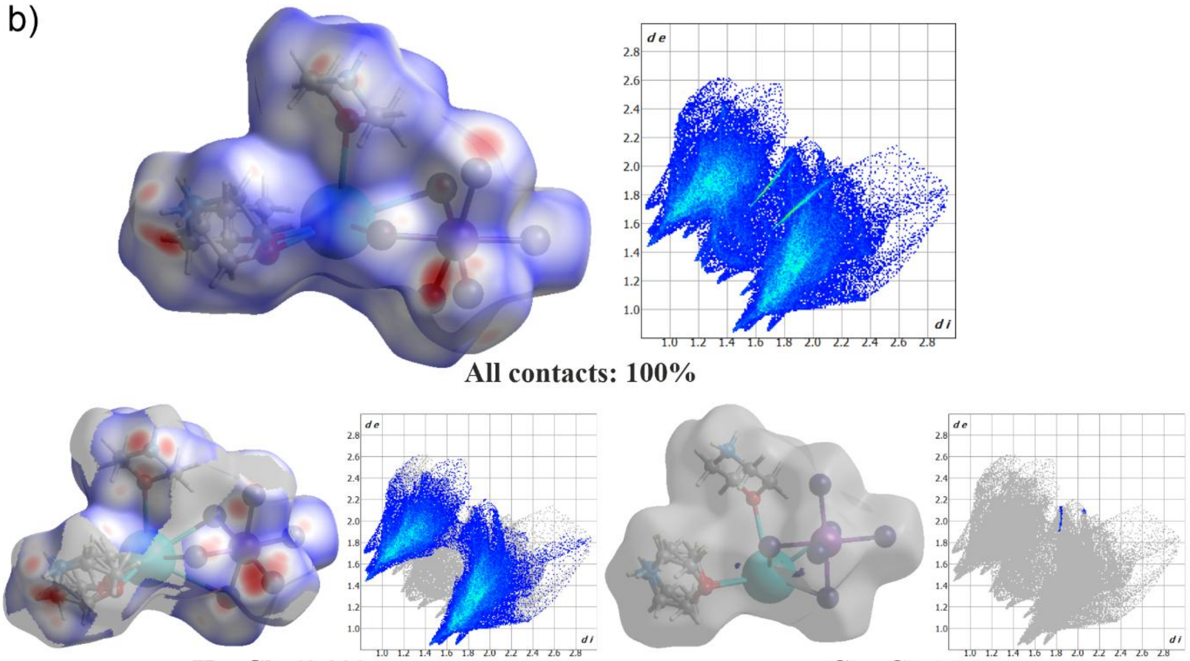

All contacts: 100%
H···Cl: 68.3%
Cl···Cl: 0.3%

c)

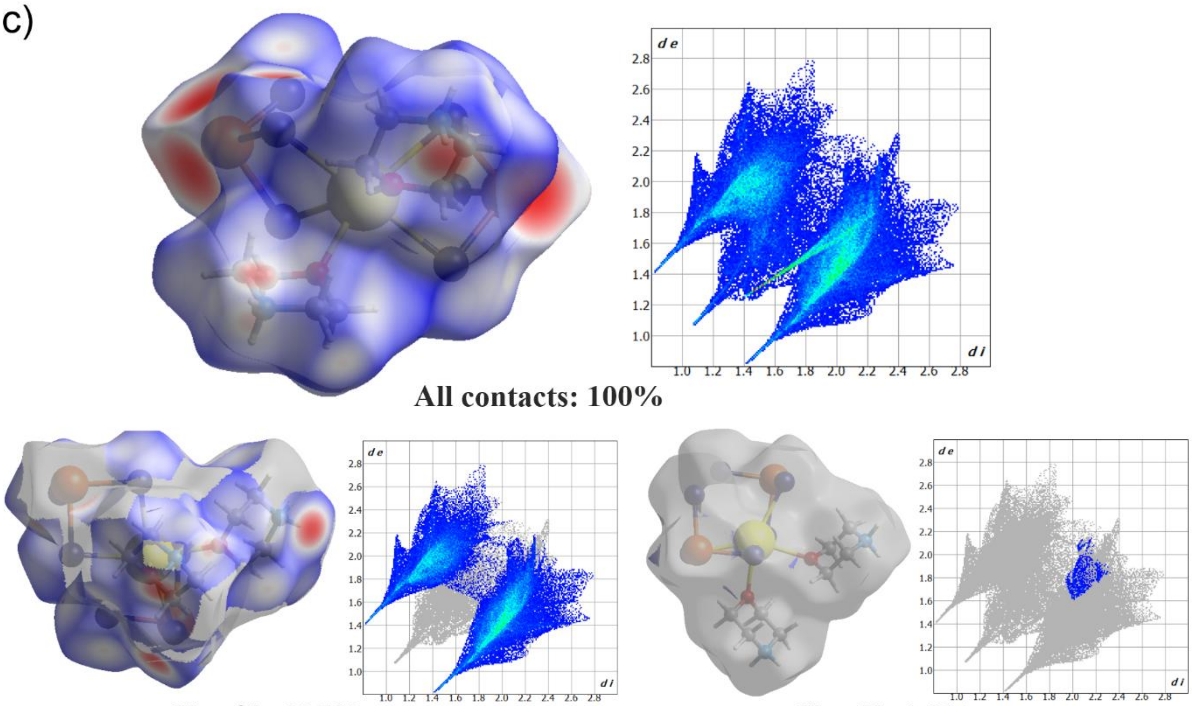

All contacts: 100%
H···Cl: 66.7%
Cl···Cl: 1.0%

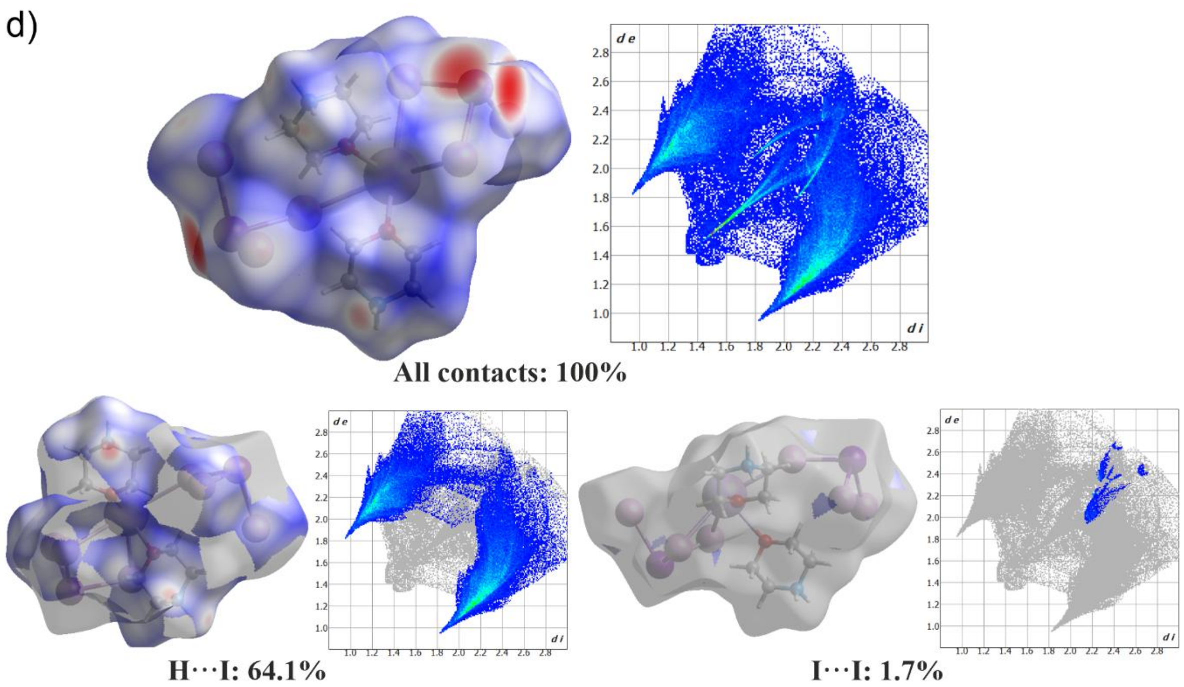

Figure S10. Hirshfeld surface of compounds **1**–**4** (a-d) mapped over d_{norm}, with corresponding 2D fingerprint plots showing all interactions and highlighting the dominant H···Cl (for **1**–**3**) and H···I (for **4**) contacts. The next most significant contribution to the total Hirshfeld surface arises from H···H interactions (14.8, 16.6, 19.3, and 14.7% for **1**–**4**, respectively), visible as diffuse areas in the central region of the fingerprint pots.

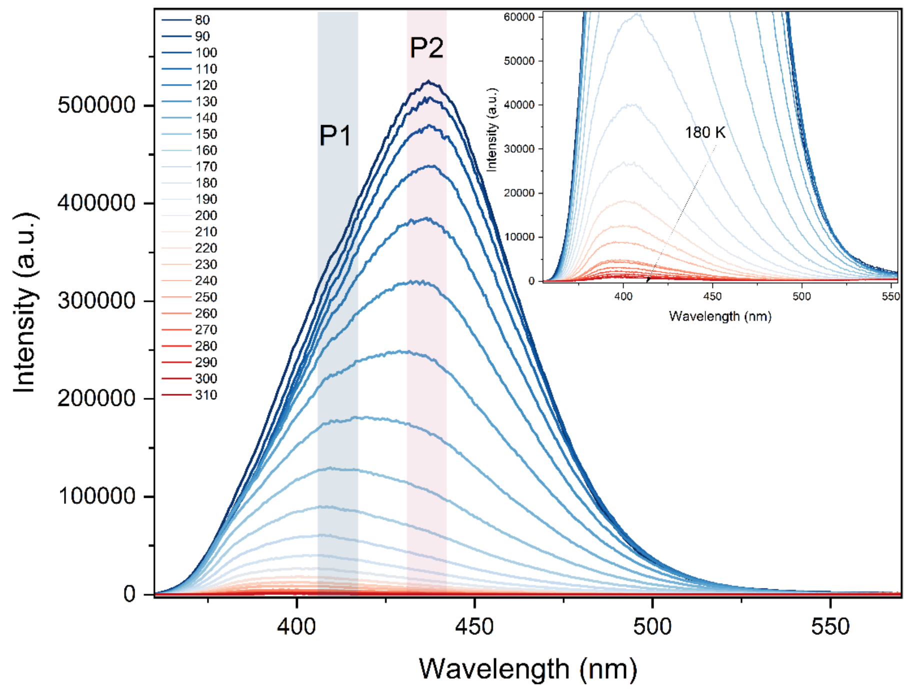

Figure S11. Temperature-dependent emission spectra recorded at various temperatures of **1**. The inset shows a magnified section of the spectrum. The red and blue areas correspond to the emission peaks from TRES.
